# A Flexible Wearable Multisensing Patch Integrating SWCNT-PtNPs Nanocomposites for Non-Invasive Clinical Biomarkers Monitoring in Sweat

**DOI:** 10.3390/polym18172150

**Published:** 2026-09-02

**Authors:** Lucian-Gabriel Zamfir, Petru Epure, Ioana Cătălina Gîfu, Iuliana Răut, Mariana Constantin, Cristina Firincă, Nicoleta-Olguța Corneli, Mihaela Doni, Ana-Maria Gurban

**Affiliations:** 1 Department of Biotechnology, National Institute for Research and Development in Chemistry and Petrochemistry-ICECHIM, 202 Spl. Independentei, 060021 Bucharest, Romania; 2 EPI-SISTEM SRL, Livezii 17A, 505600 Săcele, Romania; 3 National Institute for Medical-Military Research and Development Cantacuzino, 103 Spl. Independentei, 050096 Bucharest, Romania

**Keywords:** wearable, multisensing, biomarkers, fullerenol, nanocomposites

## Abstract

The integration of enzyme-loaded polymeric matrices with carbon-based nanomaterials and metallic nanoparticles into wearable multisensing patches, coupled with miniaturized portable detection devices, enables real-time, highly sensitive, and simultaneous monitoring of key clinical biomarkers (e.g., glucose, lactate, and H_2_O_2_) in clinical and point-of-care applications. Multiplex biosensors were fabricated by modifying screen-printed carbon paste electrodes (SPEs) with different composite nanomaterials based on carbon nanomaterials such as multi-walled carbon nanotubes (MWCNTs), single-walled carbon nanotubes (SWCNTs), or fullerenol (FL), the redox mediator Prussian Blue, and platinum nanoparticles (PtNPs). Chitosan and sol–gel polymer matrices were used to immobilize the enzymes glucose oxidase (GOx) and lactate oxidase (LOx), thus ensuring not only increased sensitivity and operational stability, but also high specificity for biomarker detection (glucose and lactate). Among the nanomaterials used for the development of multiplex biosensors, the SWCNT-PtNP composite was highlighted by electrochemical studies as having a significantly superior electrocatalytic activity toward the reduction of H_2_O_2_. This reaction occurs at a low applied potential of only −0.2 V vs. Ag/AgCl, achieving a specific sensitivity of 224.6 mA·M^−1^·cm^−2^, over a concentration range of 0.07 to 28.26 mM, and a detection limit of 3.2 μM. When functionalized with enzymes, SWCNT-PtNP-based biosensors exhibit improved conductivity, allowing the detection of glucose and lactate at a potential of −0.05 V vs. Ag/AgCl. The specific sensitivities obtained are 20.25 mA·M^−1^·cm^−2^ for glucose and 94.76 mA·M^−1^·cm^−2^ for lactate, and the corresponding detection limits are 23.6 μM and 5.0 μM, respectively. Finally, a wearable patch integrating the multiplex (bio)sensor with a portable potentiostat enabled simultaneous, sensitive, and selective detection of glucose, lactate, and H_2_O_2_ in sweat samples.

## 1. Introduction

Clinically relevant analytes, such as hydrogen peroxide, glucose, and lactate, are key biomarkers for a wide range of pathological conditions, including inflammation [1], diabetes [2], and sepsis [3]. Beyond their well-established diagnostic significance, these analytes also provide valuable information on underlying physiological processes in healthy individuals. Glucose reflects metabolic homeostasis, lactate is an indicator of exercise intensity and muscle metabolism, while hydrogen peroxide is associated with cellular redox balance and oxidative stress. Therefore, continuous monitoring of these analytes is valuable for preventive healthcare, allowing for the assessment of physical performance and recovery, as well as early detection of metabolic or inflammatory changes before the onset of overt disease [4].

Among biological fluids, sweat represents an attractive medium for monitoring these biomarkers due to its ease of noninvasive sampling and its compatibility with wearable electrochemical (bio)sensors, which allow continuous health monitoring in healthy subjects, athletes, and patients, without the inconvenience or risks associated with repeated blood sampling. This has led to a growing demand for bioanalytical platforms capable of rapid, sensitive, and selective detection of clinically relevant biomarkers directly in sweat.

However, the design of wearable sweat (bio)sensors must take into account several physiological and technical constraints. First, as the concentrations of target analytes in sweat are considerably lower and more variable than in blood, glucose typically ranges between 10 and 200 μM, while lactate, reflecting metabolic and exercise state, ranges between 2 and 30 mM, respectively [5]. The pH of sweat fluctuates between 5 and 7 depending on the secretion rate, hydration, and individual physiology [6], thus affecting both sensor stability and enzyme activity. Other operational challenges that need to be addressed to ensure reliable and long-term monitoring of biomarkers are biofouling (accumulation of biological material on the sensor surface) and bioreceptor instability following prolonged contact with the skin. Polymeric matrices are valuable in sweat biosensor development, as they provide biocompatibility and mechanical stability while ensuring bioreceptor protection [7]. Combining bioreceptor polymeric layers with nanomaterials, such as carbon allotropes (single- and multi-walled carbon nanotubes, fullerenes, etc.) and metallic nanoparticles, and integrating them into wearable patches connected to miniaturized portable detectors enables real-time, sensitive, and simultaneous monitoring of biomarkers for clinical and point-of-care applications [8]. In the development of such biosensors, H_2_O_2_ functions both as a biological analyte and as the main product of the enzymatic degradation of glucose and lactate by oxidase enzymes (GOx and LOx). Consequently, the sensitive detection of H_2_O_2_ at low applied potentials remains a key objective in biosensor development.

Carbon allotropes are among the most promising nanomaterials used in biosensor development. Single-walled carbon nanotubes (SWCNTs), for instance, exhibit conductive and electrocatalytic properties that enhance sensor sensitivity toward H_2_O_2_ [9], while fullerenol (FL) offers a functionalized, hydrophilic surface that confers biocompatibility [10] and antifouling properties [11] both highly desirable characteristics for wearable biosensors.

Metal nanoparticles and redox mediators are frequently combined with carbon nanomaterials to form nanocomposites that enhance working electrode properties by synergistically integrating the individual advantages of each component. Metal nanoparticles such as gold, silver, and platinum nanoparticles (PtNPs) are widely used for their electrocatalytic activity and large surface area, which provide numerous active sites and facilitate the sensitive detection of low analyte concentrations [12]. Their electron transfer properties in H_2_O_2_ detection are further enhanced when combined with other conductive materials, such as SWCNTs [13]. Prussian Blue (PB) is another notable example, acting as a low-potential redox mediator that functions as an artificial peroxidase by facilitating hydrogen peroxide reduction [14,15,16].

Polymeric matrices also play an essential role as enzyme immobilization matrices, preserving enzymatic activity while providing structural stability [17]. Chitosan (CS), a natural biopolymer, is widely used for bioreceptor immobilization owing to its excellent film-forming ability, non-toxicity, and biocompatibility [18]. The electrochemical and mechanical properties of CS can be further improved by combining it with carbon allotropes such as carbon nanotubes [19] and graphene [20], which enhance electrocatalytic activity and electron transfer rates. Similarly, incorporating metallic nanoparticles alongside CS can improve sensor sensitivity and selectivity [21,22]. As a cationic polysaccharide, CS contains numerous protonated amino groups (−NH_3_^+^) giving it a positive charge in acidic media such as human sweat (pH 5–7). This favors electrostatic interaction with negatively charged biomolecules, such as glucose oxidase (GOx, isoelectric point 4.2) [23], thereby improving enzyme loading and long-term stability.

Sol–gel (SG) matrices, formed through the condensation of silanol precursors such as tetraethyl orthosilicate or tetramethyl orthosilicate, offer a versatile matrix for immobilizing biomolecules in electrochemical biosensing applications. Their facile preparation allows tunable porosity, yielding mechanically robust matrices capable of encapsulating enzymes and other bioreceptors while preserving their catalytic activity and allowing efficient analyte diffusion.

GOx was the first enzyme used in electrochemical biosensors, catalyzing the oxidation of glucose to gluconic acid while generating hydrogen peroxide as a byproduct. First-generation glucose biosensors therefore relied on direct glucose detection via H_2_O_2_ oxidation; however, this approach required high applied potentials (+0.6 V vs. Ag/AgCl) and was susceptible to interference from other oxidizing species such as ascorbic [24] and uric acids [25]. Second-generation glucose sensors address these limitations by incorporating redox mediators or electrocatalytic materials, such as metal nanoparticles [26], redox polymers [27] or carbon nanotubes [28], to facilitate electron transfer from H_2_O_2_ (generated by GOx) to the working electrode at lower potentials. A similar strategy has been applied to LOx-based biosensors for the indirect detection of lactate via H_2_O_2_ reduction [29].

Polymer nanomaterial composites represent a promising strategy for addressing the key challenges of (bio)sensing in sweat. Conductive nanomaterials enhance sensitivity, while redox mediators lower the required overpotential, reducing interference from non-specific redox-active species. Hydrophilic polymeric matrices, such as chitosan and sol–gel, further contribute to bioreceptor stability. Notably, CS has been shown to form stable layers that encapsulate bioreceptors while also mitigating biofouling by reducing protein adsorption and bacterial adhesion.

Recent advances in wearable sensing technologies have demonstrated flexible tactile sensors [30] and electromechanical characterization of fabric-based strain sensors under deformation [31], highlighting the importance of mechanical flexibility in wearable device design. Polymeric matrices have also been explored in the context of smart material fabrication for sensor functionalization [32].

While individual nanocomposites based on SWCNTs [33] or PB combined with other carbon nanomaterials [34,35] have been widely reported for multiplexed electrochemical biosensing of sweat biomarkers, systematic comparative studies between fundamentally different electrocatalytic platforms such as SWCNT-PtNP and FL-PB nanocomposites for simultaneous detection of glucose, lactate, and H_2_O_2_ in sweat remain few. Most reported wearable multiplex biosensors focus on a single nanocomposite architecture without evaluating how the choice of the electrocatalytic platform influences the detection mechanism, sensitivity, and selectivity [35,36].

**Scheme 1:**
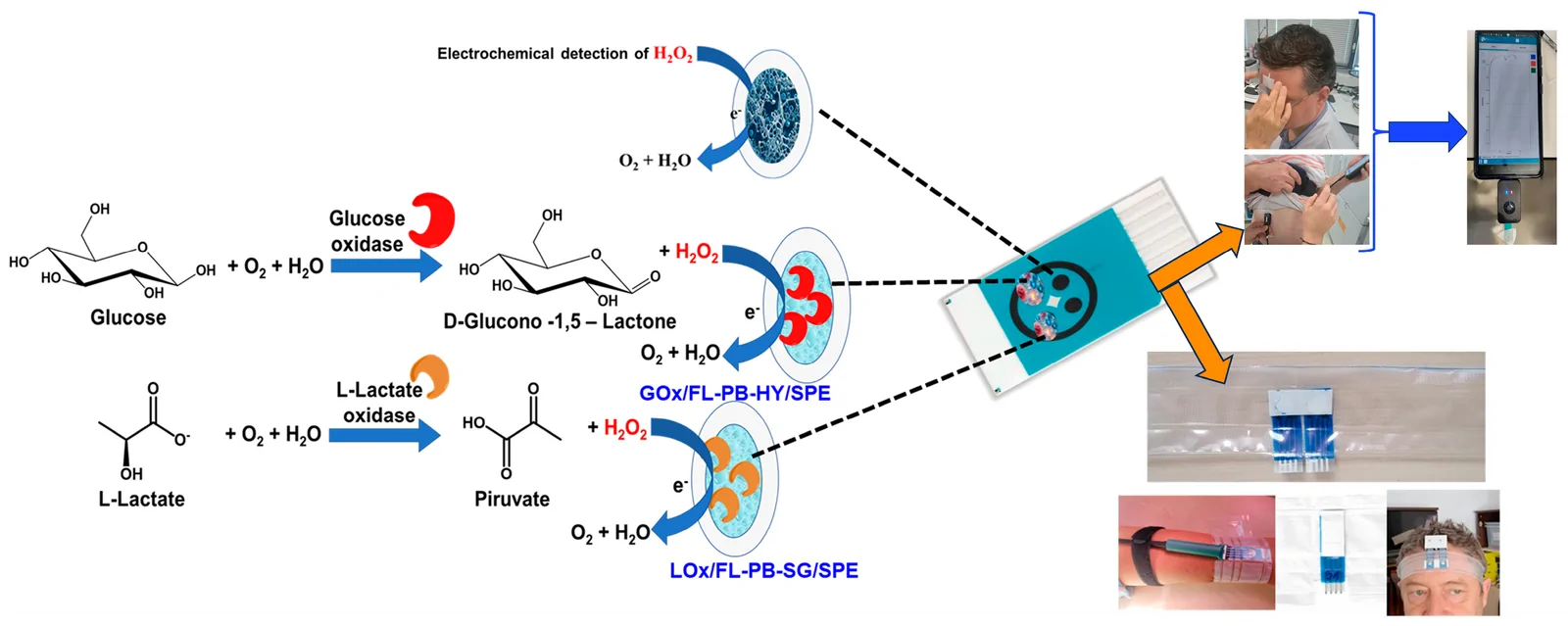
Schematic representation of a multisensing wearable patch for simultaneous analyte detection.

This work addresses this gap by developing a multiplexed electrochemical platform based on distinct nanocomposite materials for the simultaneous, sensitive, and selective detection of three clinically relevant analytes, hydrogen peroxide, glucose, and lactate, in sweat (Scheme 1). To this end, we compared the performance of screen-printed sensors modified with two nanocomposites, FL-PB and SWCNT-PtNP, for hydrogen peroxide detection, and subsequently for glucose and lactate biosensor development. To date, few studies have directly compared the performance of FL-PB and SWCNT-PtNP for H_2_O_2_ detection, particularly regarding their electrocatalytic activity across different pH conditions, an important consideration given the pH variability of sweat. Composite nanomaterials based on the carbon allotropes FL and SWCNTs, combined with the redox mediator Prussian Blue and platinum nanoparticles, respectively, were used to modify screen-printed electrodes. The electrochemical properties and sensitivity of both nanocomposites toward hydrogen peroxide reduction were systematically compared. SWCNT-PtNP exhibited the highest sensitivity and lowest detection limit for H_2_O_2_ and was therefore selected for the development of glucose and lactate biosensors. These biosensors were obtained by immobilizing GOx and LOx enzymes within CS and SG-based matrices, respectively, and were subsequently integrated into wearable patches for the detection of three clinical biomarkers in both artificial and real samples, using a portable and miniaturized potentiostat.

## 2. Materials and Methods

### 2.1. Reagents

Single-walled carbon nanotubes (SWCNT, 0.78 nm average diameter, ≥95% carbon basis), polyhydroxylated fullerenes (FL-fullerenol, C60(OH)_n_·mH_2_O, n > 40, m > 8), low molecular weight chitosan (CS, MW = 50–190 kDa), sodium acetate, acetic acid, sodium phosphate dibasic dihydrate (Na_2_HPO_4_·2H_2_O), potassium phosphate monobasic (KH_2_PO_4_), NaCl, NH_4_OH, KCl, potassium ferricyanide (K_3_Fe(CN)_6_), potassium ferrocyanide (K_4_Fe(CN)_6_), iron(III) chloride (FeCl_3_), tetramethyl orthosilicate (TMOS), methyl-trimethoxysilane (MTMOS), polyethylene glycol 550 (PEG550), glucose, sodium L-lactate, ascorbic acid, citric acid, uric acid, urea, and glucose oxidase (GOx, EC 1.1.3.4, from *Aspergillius niger*, type VII), were provided by Sigma–Aldrich (Darmstadt, Germany) and were of analytical grade. Multi-walled carbon nanotubes (MWCNTs) were obtained from Future Carbon GmbH, Bayreuth, Germany. Platinum nanoparticles (PtNPs, 2 mg/mL aqueous dispersion, 5 nm mean diameter) were obtained from Metrohm, Herisau, Switzerland. Lactate oxidase (LOx, 111 UI/mg) was purchased from Sorachim, Lausanne, Switzerland.

Electrochemical measurements were performed using 0.1 M acetate buffers for pH values ranging from 4 to 5.5, and 0.1 M phosphate-buffered saline (PBS) for pH values between 6 and 8. Hydrogen peroxide, glucose, and lactate solutions were freshly prepared in ultrapure water (18.25 MΩ·cm) immediately prior to use.

The artificial sweat solution was prepared using concentrations of 20 g/L NaCl, 17.5 g/L NH_4_OH, 5 g/L acetic acid, and 15 g/L sodium lactate [37], and the pH was adjusted to 5.5.

### 2.2. Apparatus and Measurements

A µStat 4000 Multi Potentiostat/Galvanostat was used for cyclic voltammetry and amperometric studies, operating with DropView 8400 software v3.7421B0615 for data acquisition. The electrochemical impedance spectroscopy (EIS) measurements were carried out with a portable μStat-i400s (bi)potentiostat/galvanostat in the frequency range from 100 kHz to 0.1 Hz, with a potential amplitude of 5 mV, at an open-circuit potential (OCP), in a solution of 5 mM Fe(CN)_6_^3−/4−^ in 0.1 M KCl. Commercial screen-printed electrodes (SPEs) made of carbon paste on ceramic and PVC supports and provided by Metrohm–Dropsens, Llanera, Spain (DRP-110, DRP-X1110) were used in different configurations, consisting of one, two, or three carbon paste working electrodes (Ø = 4 mm, A = 0.1256 cm^2^), a graphite counter electrode, and a silver pseudo-reference electrode.

The morpho-structural characterization of the nanomaterial-based sensors was performed using scanning electron microscopy (SEM) and Fourier transform infrared (FTIR) spectroscopy. The morphology of the modified sensor surfaces was investigated with a Hitachi SU-70 scanning electron microscope (Hitachi, Chiyoda City, Japan), equipped with an energy-dispersive X-ray analysis (EDX/EDAS) module and an Ultra-Dry Thermo Fisher Scientific detector (Thermo Fisher Scientific, Waltham, MA, USA), operating at an accelerating voltage of 4.6 kV. The structural characterization of the nanomaterials was carried out using a Bruker Tensor 37 FTIR spectrometer (Bruker, Ettlingen, Germany).

### 2.3. Preparation of Functionalized Sensors

Screen-printed carbon paste electrodes (SPEs) were modified with different carbon allotropes and nanocomposites based on FL, SWCNTs, MWCNTs, PtNPs, chitosan, and sol–gel matrices.

The SWCNT-PtNP and MWCNT-PtNP nanocomposites were prepared by sonicating a mixture of 200 µL of carbon nanotube dispersion (1 mg/mL) and 100 µL of PtNP suspension for one hour, followed by drop-casting 10 µL of the resulting mixture onto the working electrode surface. The modified sensors were dried in an oven at 65 °C, cooled to room temperature, and stored in the dark. The FL-PB-based electrochemical sensors were obtained by preparing a 2 mg/mL FL solution, mixing it with the redox mediator Prussian Blue (PB), and drop-casting 10 μL of the resulting mixture onto the working electrode surface. The PB mediator was synthesized in situ by direct precipitation onto FL by mixing FL with 0.1 M K_3_Fe(CN)_6_ and 0.1 M FeCl_3,_ each prepared in 0.01 M HCl (1:1 *v*/*v*), followed by sonication for 30 min. The modified sensors were dried in an oven at 65 °C and stored at room temperature in the dark.

### 2.4. Preparation of Bioreceptor Polymeric Layers for Biosensing

A chitosan (CS) polymeric matrix was used for GOx immobilization. GOx was mixed 1:1 with a 0.1% CS solution prepared in 2% acetic acid. The sol–gel (SG) matrix used for LOx immobilization was prepared by mixing the precursors TMOS and MTMOS in a 1:3 ratio. Following hydrolysis at 4 °C for 6 h, the enzyme was mixed with the SG solution (1:1 *v*/*v*), and 10 µL of the resulting mixture was deposited onto the sensor surface. The biosensors were stored at 4 °C. The enzyme loading was 8.11 IU/electrode for GOx-CS/FL-PB/SPE biosensors, and 3.28 IU LOx/electrode for LOx-SG/FL-PB/SPE biosensors.

### 2.5. Fabrication of Flexible Dual-Working Sensors for Wearable Patch Integration

Dual-working electrode electrochemical sensors were fabricated for integration into the wearable patch, with each working electrode designed to be functionalized independently according to the target analyte. This design yields a multi-sensor patch suitable for a range of applications, while also allowing the electrodes to be exchanged between measurement sessions to accommodate different application scenarios. Electrode fabrication was optimized with respect to the following parameters:

*Substrate.* Both flexible and rigid substrates were considered, with flexible substrates being more suitable for wearable applications, thus allowing the sensors to be adapted to different connection configurations and incorporated into the multi-sensor patch, while rigid substrates are more suitable for laboratory use or field applications where the shape of the sensor should not change during measurement. The use of flexible substrates (plastic, paper, or other flexible materials) requires the addition of polymeric binders to improve ink adhesion.

**Figure 1:**
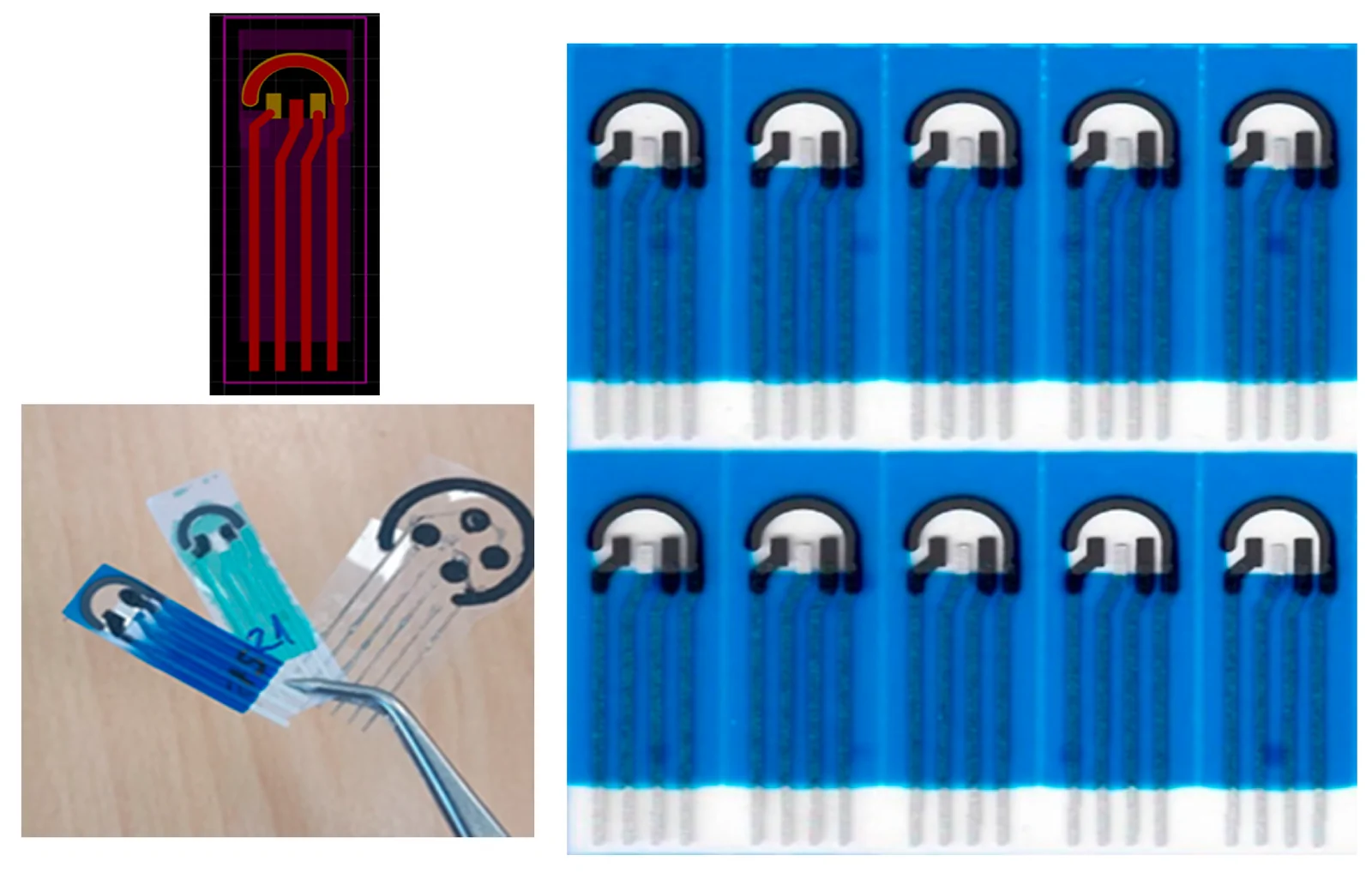
Optimized screen-printed configuration of flexible multi-sensors.

*Ink.* Conductive inks (usually carbon-, silver-, gold-, or platinum-based) were used alongside dielectric (insulating) inks. Conductive inks must exhibit low resistivity after thermal curing. Precise control of the printed pattern geometry for successive layers was essential to avoid misalignment between the silver and carbon layers during screen printing (Figure 1), as such defects significantly affect the electrochemical measurement and reduce the sensitivity of the sensor. The dielectric ink was used to isolate the connection tracks between the connector and the measurement area and to precisely define this area. This required an appropriate viscosity and adequate drying to prevent it from fusing with the paper backing layer.

*Pretreatment.* Thermal, electrochemical, or plasma pretreatments were applied. Thermal polishing was performed after each screen printing layer to fix the ink to the substrate and previously printed layers, at temperatures between 110 °C and 140 °C, depending on the ink composition and the type of substrate. After final polymerization, an electrochemical pretreatment was preferably applied to remove residual compounds left on the surface from the screen printing process, and in some cases plasma treatment was used.

The initial electrode design was developed using CAD Easy EDA V6.5.34 software (Figure 1) and tested on a simple prototype produced in-house by 2D printing on a Voltera V-One system. After eliminating design flaws and subsequent manufacturing defects, a larger batch of approximately 200 defect-free screen-printed electrodes was produced (Figure 1, right).

The electrodes were fabricated on a PVC substrate to ensure easy adaptation to the target application. Following quality evaluation, the electrodes were integrated into several types of wearable devices.

### 2.6. Design and Development of the Multiplex Miniaturized Potentiostat

**Figure 2:**
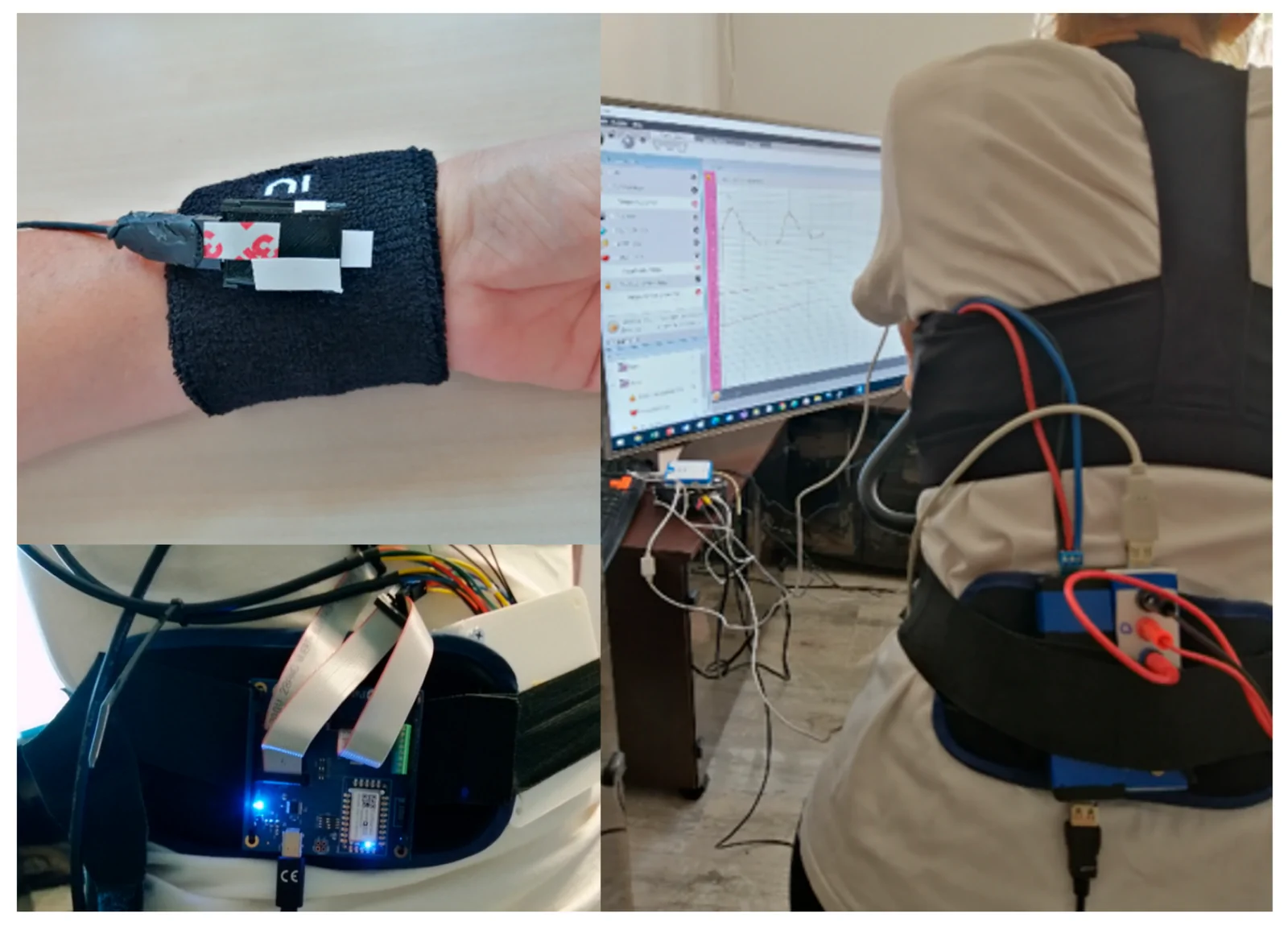
Multiplexer electrochemical measuring device (**bottom left**), sensing patch (**top left**) and bi-potentiostat (**right**).

To highlight the specific electrochemical response of each analyte, it was necessary to develop multichannel electrochemical systems capable of portable measurements and easily adaptable to wearable patches. The portability requirement demanded compact configurations that are easy to attach to the athlete without limiting physical activity. Several functional configurations were thus developed in parallel (Figure 2).

Two configurations were developed: an initial configuration with a multiplexer, which allows sequential measurement on up to 16 electrochemical channels (voltammetry and amperometry) or simultaneous recording on 8 channels, with a sampling rate of 2–4 s to preserve signal quality (Figure 2-left) and a bi-potentiostat configuration (Figure 2-right), based on the ARM Emstat Pico microcontroller (Analog Devices, in collaboration with PalmSens, Houten, The Netherlands). Compared to the SensIT variant, the bi-potentiostat configuration features a second hardware-wired working electrode and uses 4-pin connectors dedicated to dual electrodes.

This configuration currently has no direct commercial equivalent; the closest product on the market, SensIT BT, offers only two 3-pin connectors for two independent measurement channels. The developed bi-potentiostat configuration is compatible with both screen-printed and conventional electrodes, without any usage restrictions.

### 2.7. Integration and Testing of the Microfluidic Membrane into Multi-Sensor Wearable System

The purpose of the microfluidic membrane is to capture sweat accumulated on the skin surface and to collect and transport the fluid to the electrochemical cell. The microfluidic membrane can serve as a wearable patch or can be a flat cell made using 3D-printing technology to accommodate both the electrochemical sensor and the absorbent material powered by the microfluidic membrane (Scheme 1, right). The wearable patch can be placed on the forehead, limbs, or chest. The amount of sweat collected differs depending on the location and the physical and physiological characteristics of the subjects (age, sex, metabolism).

The multi-sensory, flexible, and wearable patch was used to monitor physiological parameters and the biomarkers of interest during physical exercise using a medical bicycle (Figure 2), with these patches placed on the forehead and chest and the microfluidic membrane on the right wrist. The pulse sensor was placed on the left hand, and the recording was visible on the small screen installed on the handlebars of the medical bicycle.

The coupling of these multi-sensors was initially achieved using banana cables and a specific plug for dual electrodes. The patches are flexible, can accommodate up to 3 dual sensors, and can be placed on any part of the human body. Electrochemical multi-sensors with 2 or 4 working electrodes were prepared, which led to obtaining a multi-sensor patch that also allows the electrodes to be changed between measurement sessions so that more application variants can be addressed. A wearable patch capable of measuring the electrochemical response collected from multiple sensors located in a bracelet or band and allowing measurements during exercise activities was developed. To highlight the level of effort, a pulse sensor was also used so that electrochemical measurements were made after a period of at least 5–10 min of effort.

## 3. Results and Discussions

### 3.1. Morpho-Structural Characterizations of the Nanomaterial-Based Electrochemical (Bio)Sensors

SEM was used to investigate the surface morphology of SPE sensors modified with FL, PB, FL-PB, CS/FL-PB, SWCNTs, PtNPs, SWCNT-PtNP, and CS/SWCNT-PtNP (Figure 1).

**Figure 3:**
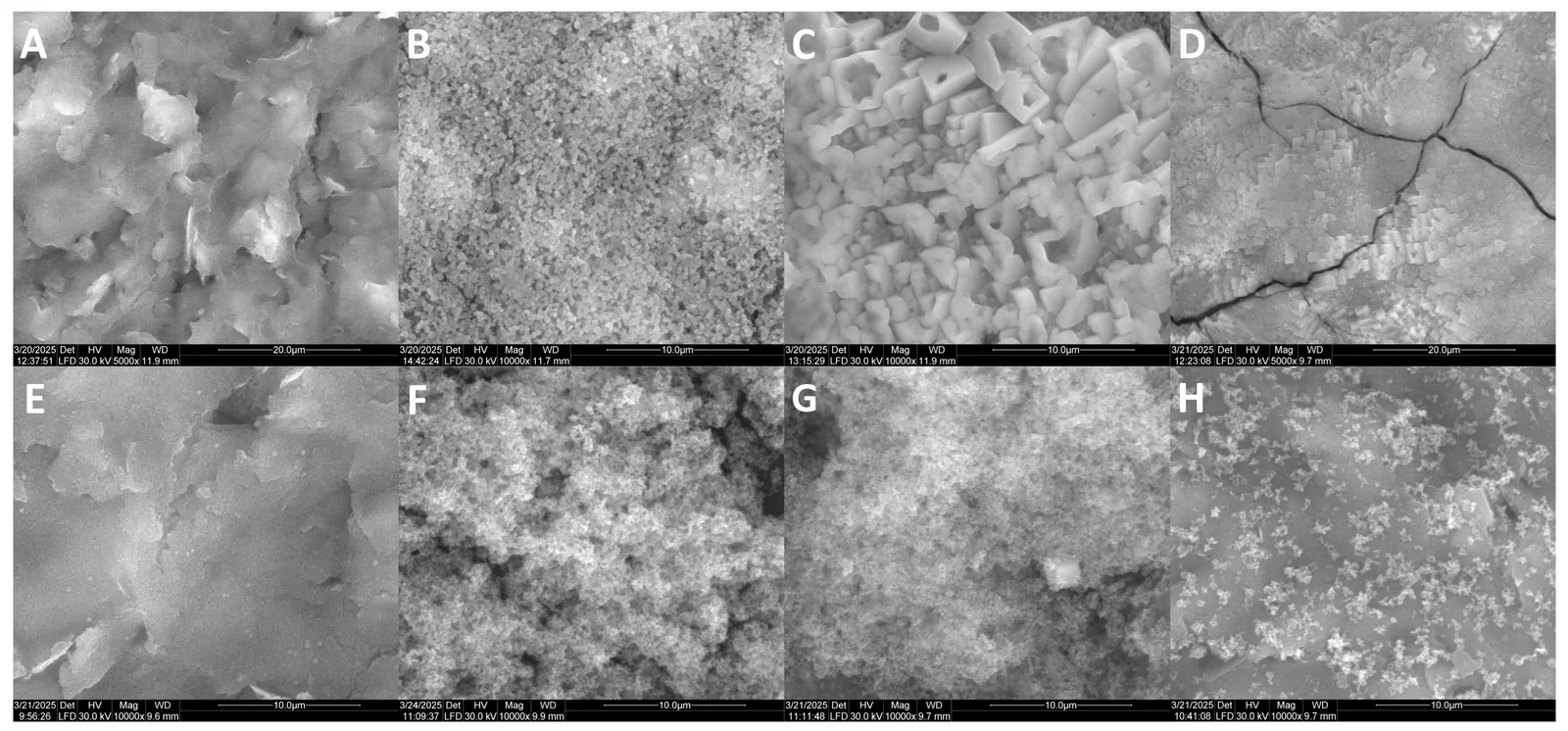
SEM images of: (**A**) FL/SPE, (**B**) PB/SPE, (**C**) FL-PB/SPE, (**D**) CS/FL-PB/SPE, (**E**) SWCNT/SPE, (**F**) PtNP/SPE, (**G**) SWCNT-PtNP/SPE, and (**H**) CS/SWCNT-PtNP/SPE.

Figure 3A shows aggregated nanosheets on the FL-modified surface, formed through hydrogen bonding between the hydroxyl groups of FL, resulting in a porous layer with non-uniform coverage. The PB/SPE sensors exhibit a granular texture, with PB deposited as nanocrystals providing uniform coverage and a rough surface, suggestive of a large electroactive surface area. In Figure 3C, PB microcrystals can be observed growing on FL aggregates, indicating that FL acts as a nucleation site for PB formation, in a manner similar to the nucleation of PB on carbon nanotubes [38]. The CS/FL-PB layers display cracks and exposed regions, which can be attributed to the brittleness of the films during and after drying. In the Supplementary Materials (Figure S1), the SEM image of SG/FL-PB confirms the effective coverage and entrapment of FL-PB within the porous SG layer.

For SWCNT/SPE, a thin and smooth SWCNT layer is observed, suggesting a uniform coating of the electrode surface (Figure 3E). PtNP/SPE displays a granular surface, consistent with dense nanoparticle coverage. The SWCNT-PtNP sensors are characterized by a porous nanostructure, with PtNPs uniformly distributed along the SWCNTs, a feature correlated with the enhanced electrocatalytic activity toward hydrogen peroxide reduction. SG/SWCNT-PtNP/SPE shows a predominantly smooth morphology, indicating entrapment of the nanomaterial layer within the SG matrix, although some cracks are also visible as a result of the drying process.

CS/SWCNT-PtNP/SPE exhibits a rough, irregular film morphology, with aggregates formed through the interaction between CS and SWCNT-PtNPs; this morphology provides a high surface area, favoring greater enzyme loading and, consequently, an enhanced biosensing signal (Figure S1C). GOx-CS/SWCNT-PtNP/SPE shows a denser, porous structure, indicating the incorporation of GOx within the CS matrix due to the electrostatic interaction between the positively charged amino groups of CS and the negatively charged sites of the GOx enzyme.

LOx-SG/SWCNT-PtNP/SPE exhibits large pores that are likely to facilitate substrate diffusion (Figure S1D). The SG matrix based on TMOS and MTMOS carries a negatively charged surface due to the presence of negatively charged silanol groups (Si-O^−^). The transition from the smooth morphology of SG/SWCNT-PtNP to the highly porous, cavity-rich structure of LOx-SG/SWCNT-PtNP (Figure S1B,D) can be explained by the electrostatic repulsion between the negatively charged SG matrix and the negatively charged LOx enzyme, which disrupts the compactness of the film.

Thus, the SEM images confirm the successful formation of SWCNT- and PtNP-based nanomaterials and their uniform distribution across the SPE surfaces, along with an increase in surface roughness indicative of a higher electroactive surface area, favorable both for electrochemical reactions and for bioreceptor immobilization. Furthermore, GOx was shown to interact strongly with the CS matrix, and LOx with the SG film, resulting in the formation of stable biosensing layers.

### 3.2. Structural Characterization of the Nanomaterials

The structural properties of the nanomaterials were characterized using FTIR spectroscopy. The full spectra of the analyzed materials in the 4000–500 cm^−1^ range are provided in the Supplementary Information (Figure S2).

**Figure 4:**
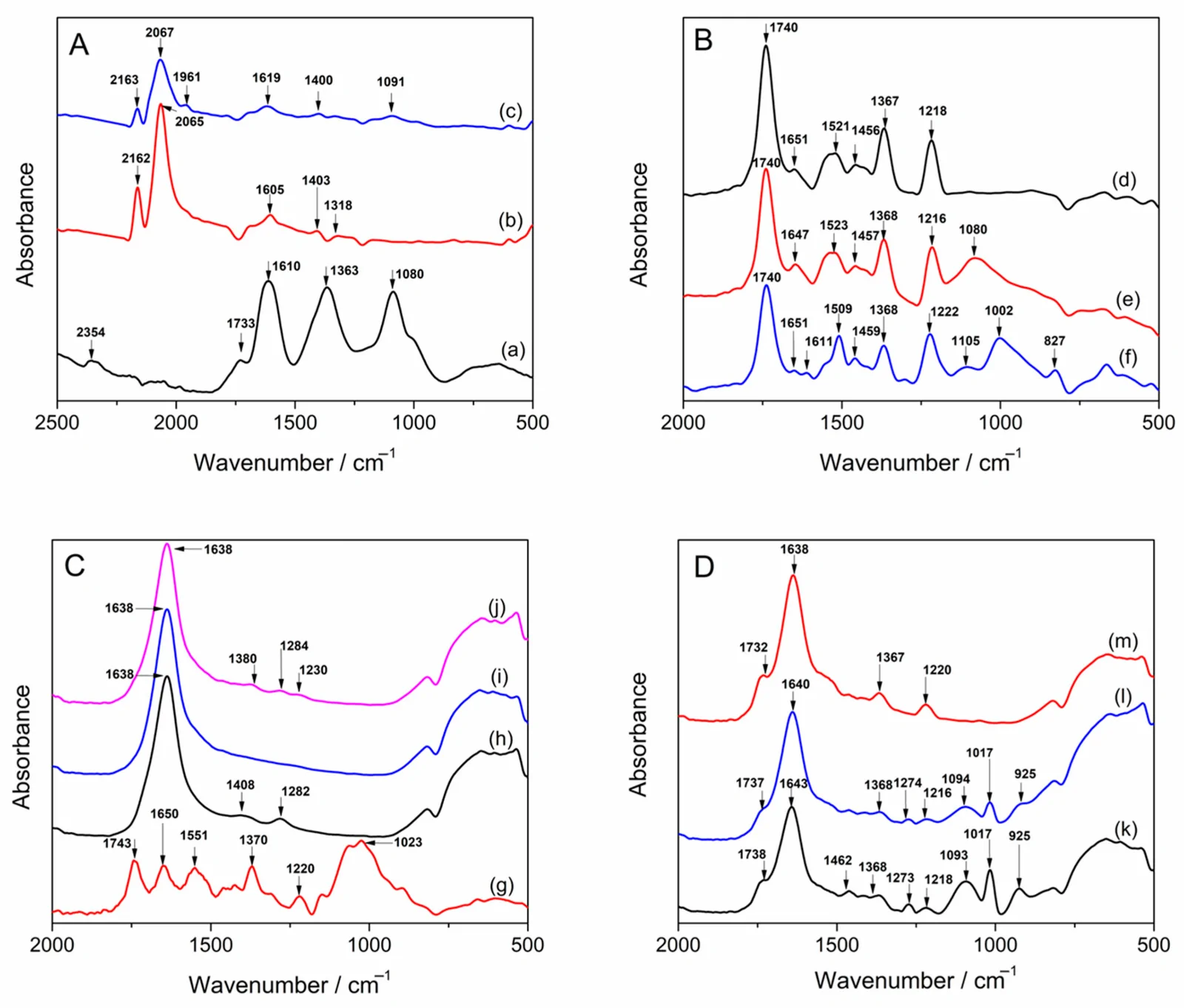
FTIR spectra for: (**A**) FL (a), PB (b), and FL-PB (c); (**B**) SWCNT (d), PtNP (e), SWCNT-PtNP (f); (**C**) CS powder (g), CS in acetic acid solution (h), GOx (i), GOx-CS (j); and (**D**) SG (k), LOx (l), and LOx-SG (m).

Figure 4A shows the FTIR spectra of FL, PB, and FL-PB. PB displays characteristic C≡N stretching bands of ferrocyanide at 2065 and 2162 cm^−1^ [39]. The spectrum of FL shows a band at 1610 cm^−1^, attributed to aromatic C=C vibrations of the fullerene carbon structure, while the bands at 1363 and 1080 cm^−1^ correspond to O-H bending and C-O stretching of the hydroxyl groups, confirming the hydroxylation of the fullerene surface [40]. In the FL-PB spectrum, bands from both precursors are present, and the C≡N band shows a small shift to 2067 cm^−1^ along with a decrease in intensity, suggesting hydrogen bonding between the -OH groups of FL and PB.

The spectra for SWCNT, PtNP, SWCNT-PtNP, and CS/SWCNT-PtNP are shown in Figure 4B. All spectra exhibit bands indicating carboxylic groups, likely corresponding either to oxidized regions on the SWCNTs or to functional groups of the stabilizer residues in the case of PtNPs. Thus, all spectra show a band near 1740 cm^−1^, attributed to C=O stretching, an asymmetric COO^−^ band at 1509–1523 cm^−1^, which may overlap with aromatic C=C vibrations, and bands at 1367–1368 cm^−1^ and 1218–1222 cm^−1^, corresponding to carboxylic C-H bending and C-O stretching, respectively. In the SWCNT-PtNP spectra, the asymmetric COO^−^ band shifts to 1509 cm^−1^, indicating an interaction between the stabilizer molecules and the carboxylic groups of the SWCNT. The PtNP spectrum shows a band at 1080 cm^−1^, attributed to C-O-C vibrations of the organic stabilizer. After mixing with SWCNTs, new bands appear at 1105, 1002, and 827 cm^−1^, associated with changes in coordination at the SWCNT-PtNP interface.

The interactions between the polymer matrices (CS and SG) and the immobilized GOx and LOx enzymes were also characterized. For most samples, the broad bands at 1638 cm^−1^ (GOx, GOx-CS, LOx), 1640 cm^−1^ (LOx-SG), and 1643 cm^−1^ (SG) are dominated by the H-O-H bending of absorbed water present in the aqueous samples, which also masks the amide I region for CS, GOx, and LOx. In Figure S2C, the spectrum of CS powder shows C-H symmetric and asymmetric stretching bands at 2921 and 2871 cm^−1^, attributed to polysaccharide CH_2_/CH_3_ vibrations. The presence of residual N-acetyl groups was confirmed by the band at 1551 cm^−1^ for N-H bending of amide II, while the band at 1370 cm^−1^ is associated with C-H bending in the glucosamine ring of CS. In CS dissolved in 2% acetic acid, these bands broaden or disappear as CS becomes ionized (−NH_3_^+^). In the CS-GOx film, distinct bands appear in the 1370, 1280, and 1230 cm^−1^ region, which may result from a combination of C-N, C-O, and C-H vibrational modes of the CS backbone together with amide III, C-N, and N-H bending modes of the protein, which become detectable once the enzyme is encapsulated within CS. Their presence in the composite, but absence in the GOx solution alone, can be attributed to polymer-protein coupling through hydrogen bonding and electrostatic interactions, which allows previously masked protein bands to become detectable.

Figure 4D shows the 2000–500 cm^−1^ region of the FTIR spectra of SG, LOx, and SG-LOx. SG exhibits siloxane (Si-O-Si) asymmetric stretching bands at 1017 and 1093 cm^−1^, together with a strong Si-OH band at 925 cm^−1^, corresponding to unbound silanol groups. This band decreases in intensity for SG-LOx, suggesting that LOx interacts with the surface silanol groups through hydrogen bonding, confirming enzyme-matrix interactions. The persistence of the siloxane bands at 1017 and 1094 cm^−1^ in the SG-LOx spectrum confirms that the siloxane network remains stable and that LOx entrapment does not alter its structure.

### 3.3. Cyclic Voltammetry Studies

**Figure 5:**
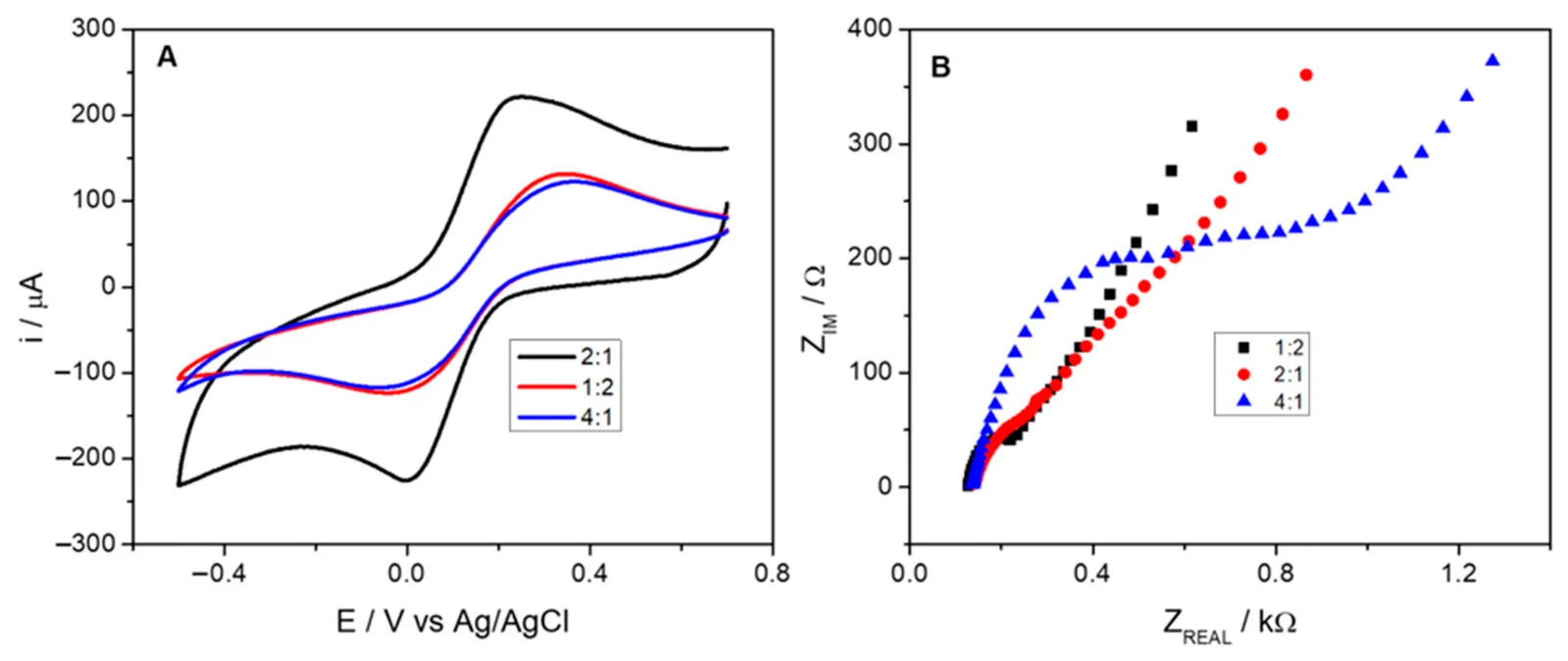
Optimization of SWCNT:PtNP volume ratio (1:2, 2:1 and 4:1). (**A**) Cyclic voltammograms recorded in 5 mM [Fe(CN)_6_]^3−/4−^, 0.1 M KCl, ν = 0.1 V/s. (**B**) Nyquist plots were recorded in the same solution (frequency range: 100 kHz–0.1 Hz, AC amplitude: 5 mV, OCP).

Prior to electrochemical characterization, the SWCNT: PtNP volume ratio was optimized by evaluating three compositions (1:2, 2:1, and 4:1, *v*/*v*) using cyclic voltammetry and EIS in 5 mM [Fe(CN)_6_]^3−/4−^(Figure 5). It can be observed that the 2:1 SWCNT:PtNP ratio produces the highest peak currents in CV and the lowest charge transfer resistance in EIS, indicating optimal electrocatalytic activity and interfacial conductivity. At lower PtNP content (1:2 ratio), insufficient electrocatalytic sites limit the current response, while excess PtNP relative to SWCNT (4:1) likely disrupts the conductive nanotube network, increasing resistance. Thus, a 2:1 ratio was selected for all subsequent (bio)sensor preparation and characterization.

The electrocatalytic properties of the SPE modified with various nanomaterials toward H_2_O_2_ reduction were studied by cyclic voltammetry, using 0.1 M PBS, pH 7, in the presence of 1 mM H_2_O_2_, by sweeping the potential between 0.6 and −0.8 V vs. Ag/AgCl at a scan rate of 0.1 V/s. The current intensity and peak potential for the reduction of 1 mM H_2_O_2_ were recorded for each modified SPE sensor.

**Figure 6:**
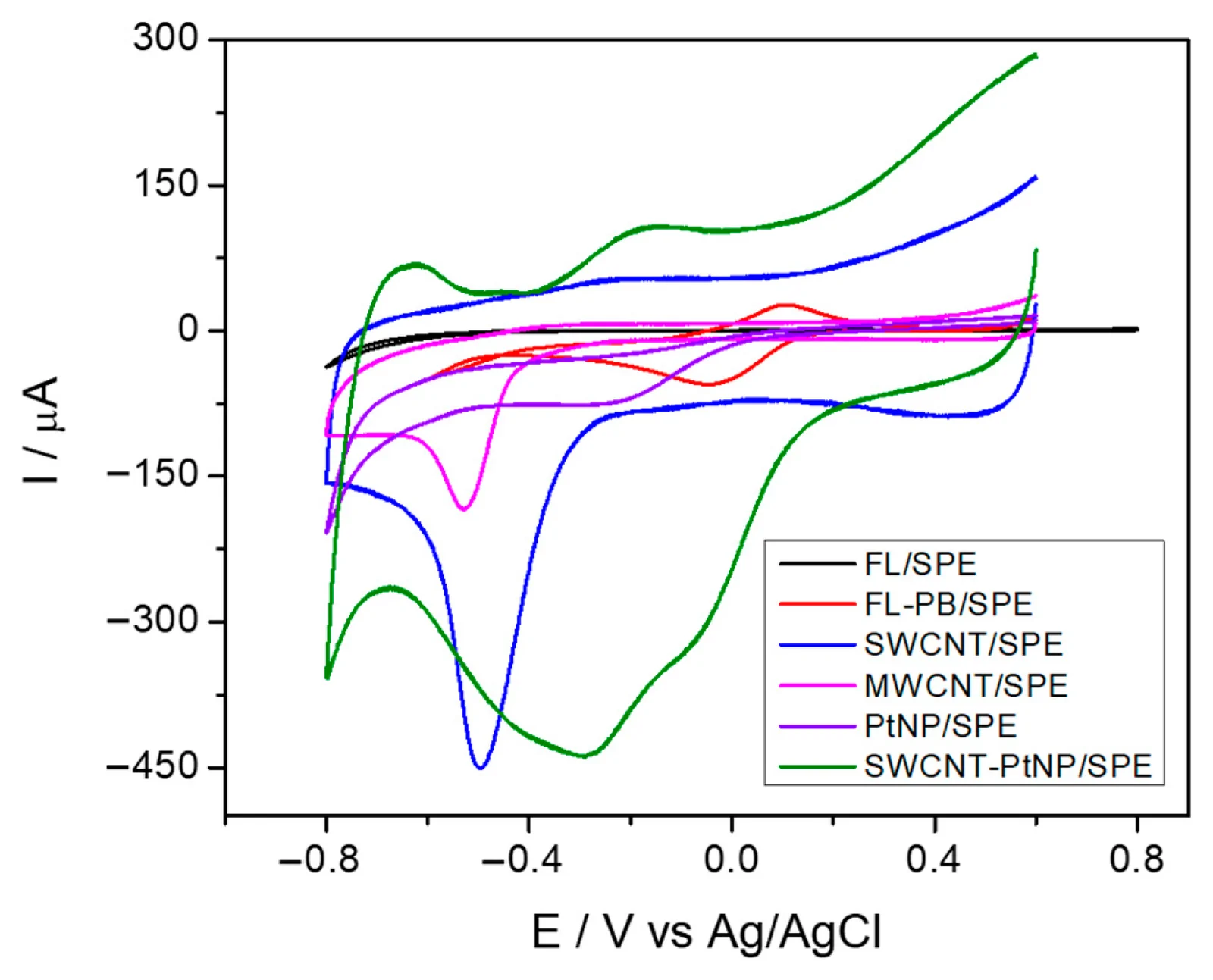
Cyclic voltammograms recorded for: FL/SPE, FL-PB/SPE, SWCNT/SPE, MWCNT/SPE, PtNP/SPE, and SWCNT-PtNP/SPE (0.1 M PBS, pH 7, 1 mM H_2_O_2_, ν = 0.1 V/s).

Figure 6 shows the CV measurements obtained for the bare SPE and for SPEs modified with FL, FL-PB, SWCNT, MWCNT, PtNP, and SWCNT-PtNP.

The cyclic voltammograms show that depositing carbon nanotubes on the SPE significantly changes the signal for hydrogen peroxide reduction. While the FL/SPE sensor showed no reduction peak, the FL-PB/SPE sensor exhibited catalytic activity toward hydrogen peroxide, due to the contribution of the redox mediator PB.

For the sensors modified only with PtNPs, hydrogen peroxide reduction occurred at a higher potential value of −0.249 V, with a cathodic peak current of −63.54 μA. For the SPE sensors modified with MWCNTs, hydrogen peroxide reduction occurred at a relatively high overpotential of −0.53 V, with a cathodic peak current of −172.9 μA. The SWCNT/SPE sensor showed a lower potential value of −0.497 V, with a higher current of −351.9 μA. The sensors modified with SWCNT-PtNP showed the lowest reduction potential, −0.23 V, and the highest reduction current, −301 μA.

Thus, SWCNTs show a higher current and lower overpotential due to their larger exposed surface area, whereas for MWCNTs, the inner walls are not accessible, resulting in a smaller surface area available for electrochemical reactions. PtNPs decrease the overpotential for H_2_O_2_ reduction but also give lower current due to a more limited electrochemically active surface area when the PtNPs aggregate. For SWCNT-PtNPs, the SWCNTs act as a support that prevents PtNP aggregation, which further enhances the electrochemical interactions.

**Figure 7:**
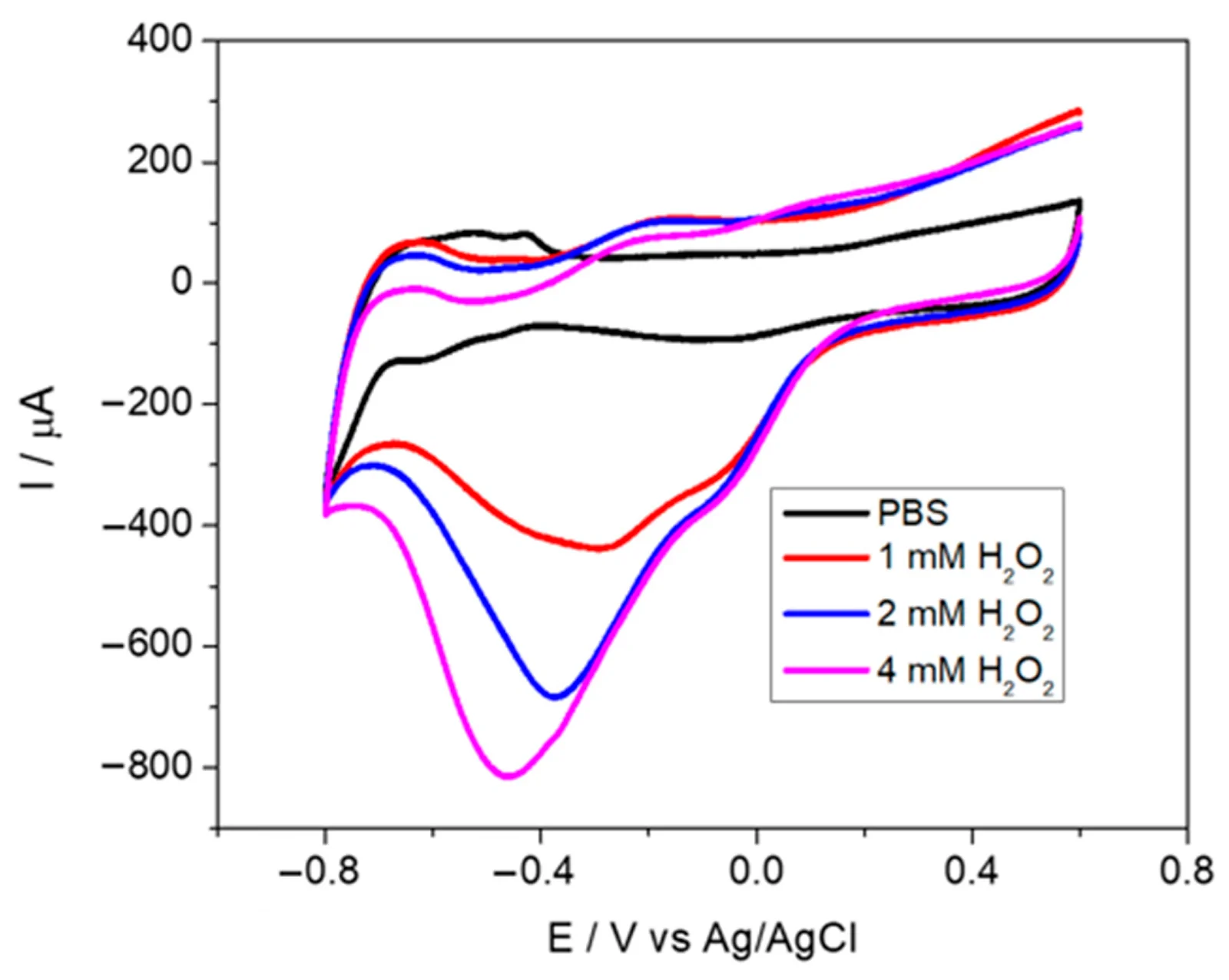
CV measurements carried out in the absence and presence of different H_2_O_2_ concentrations using the SWCNT-PtNP/SPE sensors (0.1 M PBS, pH 7, ν = 0.1 V/s).

CV measurements carried out at different H_2_O_2_ concentrations with the SWCNT-PtNP/SPE sensor (Figure 7) show an increase in the reduction peak current with increasing hydrogen peroxide concentration, from −301 µA at 1 mM H_2_O_2_ to −661 µA at 4 mM, accompanied by a shift of the reduction potential toward more negative values, from −0.287 V to −0.456 V, characteristic of a diffusion-controlled electrocatalytic process.

The CV studies demonstrate the synergistic effect of combining SWCNTs and PtNPs as a working electrode material for H_2_O_2_ sensors, with the SWCNT-PtNP-based sensors showing enhanced electrocatalytic activity toward hydrogen peroxide reduction. At the SPE surface, PtNPs facilitate the adsorption and dissociation of H_2_O_2_ into reactive oxygen species intermediates. These are then rapidly protonated and reduced, lowering the activation energy and shifting the reduction potential to less negative values compared with the bare SPE sensor. In addition, the nanoscale nature of the PtNPs increases the electroactive surface area, further enhancing the electrocatalytic currents [41].

The interaction between SWCNTs and PtNPs accelerates the electron transfer process at the sensor surface, leading to an increased cathodic peak current and a lower reduction potential. Consequently, the SWCNT-PtNP-based sensor was selected for subsequent electrochemical studies and for the development of amperometric biosensors for glucose and lactate detection.

**Figure 8:**
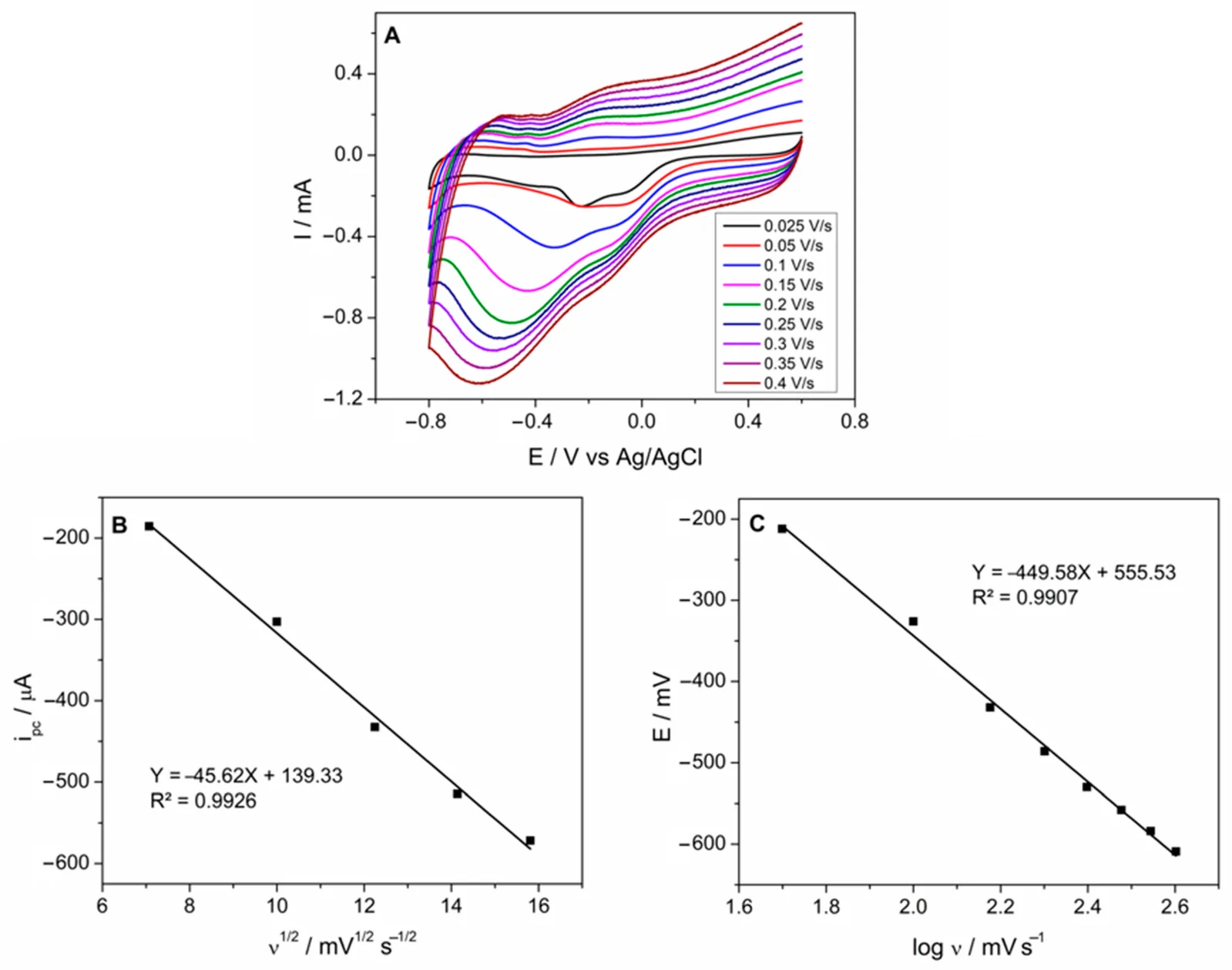
(**A**) Cyclic voltammograms recorded with SWCNT-PtNP/SPE at different scan rates (0.1 M PBS, pH 7.4, 1 mM H_2_O_2_, scan rate: 0.025;0.05; 0.1; 0.15; 0.2; 0.25; 0.35 and 0.4 V/s). (**B**) Plot of cathodic peak current (Ipc) vs. square root of the scan rate (ν^1/2^). (**C**) Plot of cathodic potential (Epc) vs. log of the scan rate (log ν).

The electrocatalytic behavior of SWCNT-PtNP/SPEs was evaluated by cyclic voltammetry at different scan rates, from 0.025 to 0.4 V/s, in 0.1 M PBS, pH 7.4, containing 1 mM H_2_O_2_ (Figure 8A).

Cyclic voltammograms performed with SWCNT-PtNP sensors showed that the cathodic peak current increases linearly with the square root of the scan rate (I_pc_ vs. ν^1/2^ plot), which is correlated with a diffusion-controlled process (Figure 8B). The cathodic peak potential (E_pc_) shifted significantly with the scan rate, as can be observed in the Epc vs. log v plot (Figure 8C), which is characteristic of a kinetically limited, irreversible electron transfer. The CV study showed that the reduction process is sensitive to changes in scan rate, consistent with a multi-electron reduction mechanism of hydrogen peroxide on Pt surfaces, involving an initial adsorption step followed by an irreversible electron transfer. These results are in agreement with the high sensitivity observed for the SWCNT-PtNP/SPE sensor, as the fast diffusion of H_2_O_2_ toward the highly conductive SWCNT network, combined with the electrocatalytic activity of PtNPs, ensures efficient charge transfer.

**Figure 9:**
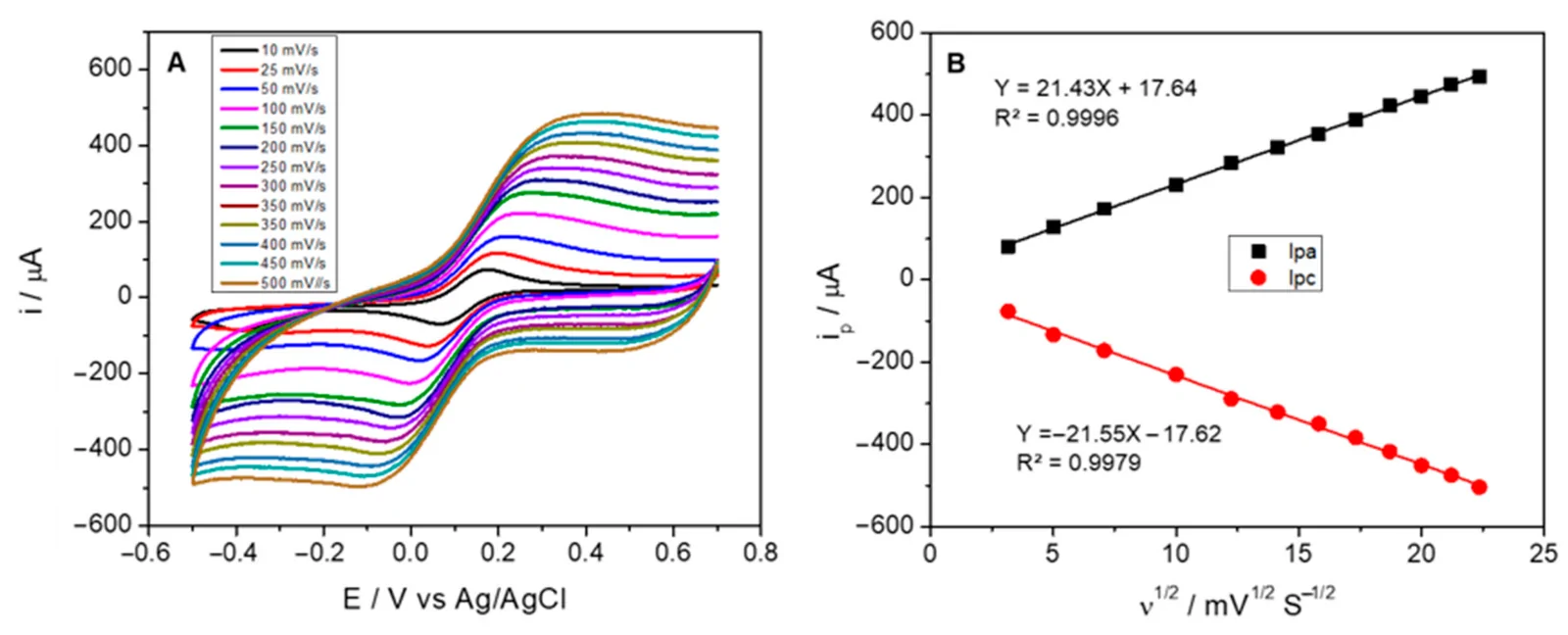
Cyclic voltammograms recorded for SWCNT-PtNP/SPE: (**A**) at scan rates ranging from 0.01 to −0.5 V/s in 5 mM [Fe(CN)_6_]^3−/4−^, (**B**) Randles-Sevcik plots of oxidation (Ipa- black) and reduction (Ipc-red) peak currents vs. the square root of scan rate (ν^1/2^).

The electroactive surface area of the modified SWCNT-PtNP/SPE sensor was calculated using the Randles–Ševčík equation ($i_{p}=2.69\;\times \;10^{5}\;n^{3/2}\;AC\sqrt{D\nu }$, where T = 25 °C and *n* = 1), using the values of the slopes that correspond to the ip vs. ν^1/2^ plots (Figure 9B) and the known value of the diffusion coefficient (D) for the [Fe(CN)_6_]^3−/4−^ redox couple (7.6 × 10^−6^ cm^2^ s^−1^). Thus, the electroactive surface area calculated was 0.186 ± 0.001 cm^2^, higher than the geometric area of the working electrode, 0.1256 cm^2^, which shows that the SWCNT-PtNP nanocomposite significantly increases the electroactive surface area.

**Figure 10:**
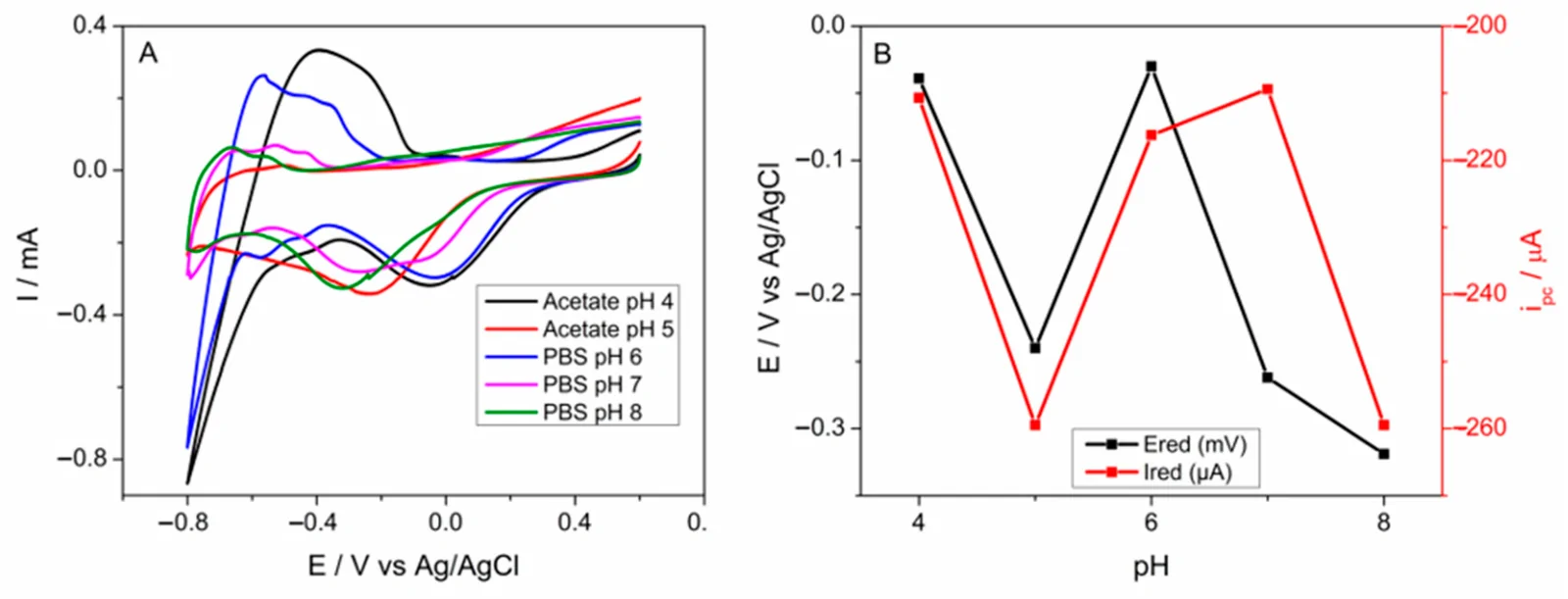
The effect of pH on the electrocatalytic behavior of SWCNT-PtNP/SPE: (**A**) cyclic voltammograms in acetate and PBS buffers with 1 mM H_2_O_2_, and (**B**) variation of reduction peak potential and current vs. pH (ν = 0.1 V/s).

The influence of the buffer pH on H_2_O_2_ detection was studied using SWCNT-PtNP/SPEs by cyclic voltammetry, in buffers with different pH values (Figure 10).

The cathodic peak potential shifts with pH, which is correlated with the proton dependence of H_2_O_2_ reduction, while the peak current intensity remains relatively stable across the pH range studied. The marked shift in potential between pH 5 and 6 is mainly due to the change in buffer types, from acetate (pH 4–5) to phosphate (pH 6, 7, 8). The adsorption and competitive blocking of the PtNP surface by different anion types lead to shifts in the voltammetric peaks.

The variation in reduction peak current intensity is not significant, and the pH studies show that the SWCNT-PtNP/SPE sensors are chemically stable across a wide pH range (4–8), confirming their suitability for a broad range of applications. At the slightly acidic pH values characteristic of sweat (pH 5–7 for real samples, pH 5.5 for artificial sweat), the SWCNT-PtNP/SPE sensors show high current intensity, between −210 and −260 µA, indicating stable and strong electrocatalytic activity, while Epc varies from −0.03 to −0.24 V. The electrocatalytic activity of PtNPs and the fast electron transfer provided by SWCNTs are maintained despite variations in buffer pH and composition.

### 3.4. Electrochemical Impedance Spectroscopy Studies

**Figure 11:**
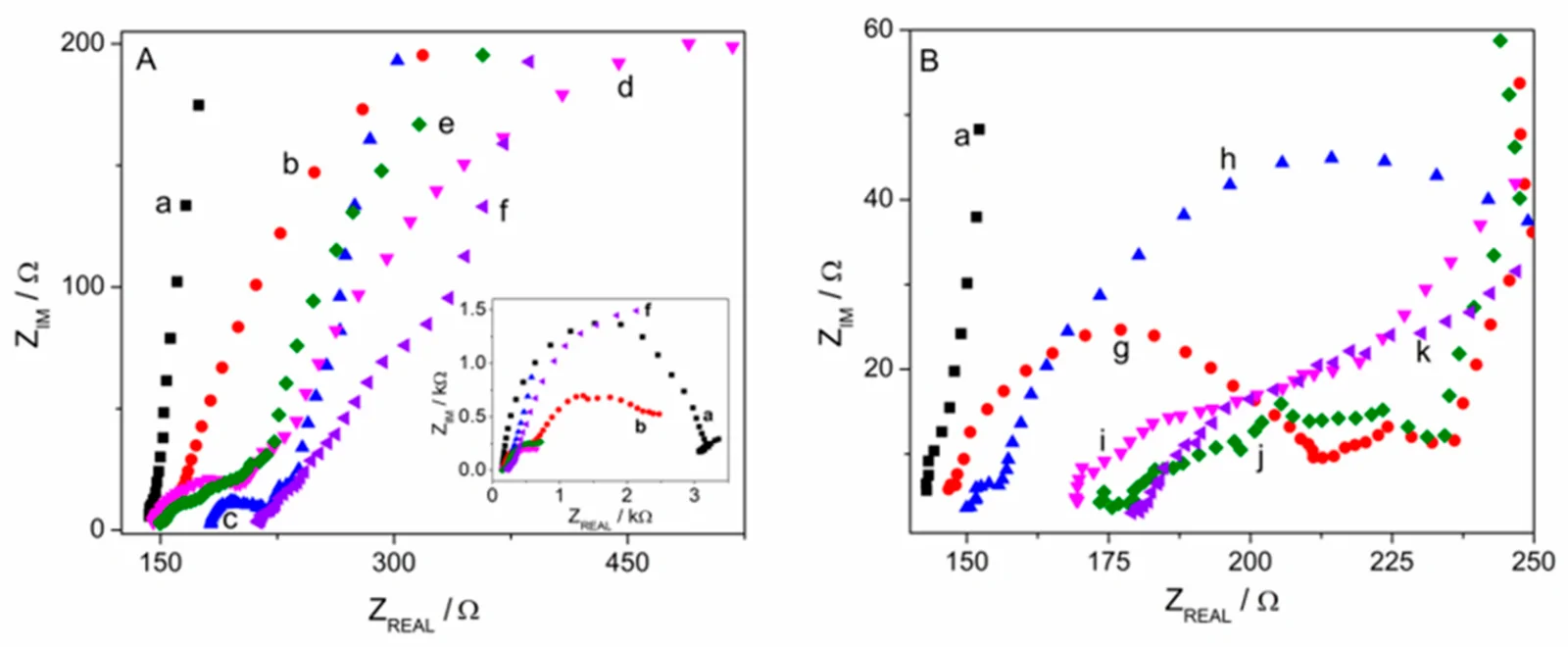
Nyquist plots for: unmodified (a) SPE; (**A**)- FL based sensors—(b) FL/SPE, (c) PB/SPE, (d) FL-PB/SPE, (e) CS/FL-PB/SPE, (f) SG/FL-PB/SPE, and (**B**) SWCNT based sensors—(g) SWCNT/SPE, (h) PtNP/SPE, (i) SWCNT-PtNP/SPE, (j) CS/SWCNT-PtNP/SPE, and (k) SG/SWCNT-PtNP/SPE. (frequency range: 100 kHz–0.1 Hz, AC amplitude: 5 mV, OCP).

EIS was used to characterize the charge transfer properties of the nanomaterial-modified sensors. The Nyquist plots were recorded for the SPE, FL/SPE, PB/SPE, FL-PB/SPE, CS/FL-PB/SPE, SG/FL-PB/SPE, SWCNT/SPE, PtNP/SPE, SWCNT-PtNP/SPE, CS/SWCNT-PtNP/SPE, and SG/SWCNT-PtNP/SPE sensors using a 0.1 M KCl solution containing 5 mM of Fe(CN)_6_^3−/4−^ as a redox probe (Figure 11). The EIS data were fitted using two Randles-type equivalent circuits, selected according to the shape of the Nyquist plots. For plots showing a single semicircle followed by a diffusion-controlled region, the circuit [R_s_(Q_dl_[R_ct_W])] was applied, where R_s_ is the resistance of the electrolyte solution, Q_dl_ is the double-layer capacitance, R_ct_ is the charge transfer resistance at the electrode surface, and W is the Warburg diffusion element. For plots showing two distinguishable semicircles, such as those of FL/SPE, FL-PB/SPE, CS/FL-PB/SPE, and SG/FL-PB/SPE, the circuit [R_s_(R_f_Q_f_)(R_ct_Q_dl_)] was applied; R_s_ retains the same meaning as above, R_f_ and Q_f_ represent the resistance and capacitance of the surface layer, respectively, and R_ct_ and Q_dl_ represent the charge transfer resistance and double-layer capacitance at the electrode/electrolyte interface. Schematic representations of both equivalent circuits are shown in Figure S3.

The deposition of nanomaterials on the SPE decreased the R_ct_ value from 2913 Ω for the bare SPE to 68 Ω for SWCNT/SPE and 88 Ω for SWCNT-PtNP/SPE (Table 1). SWCNTs provide a high electroactive surface area, while PtNPs supply catalytic sites for electron transfer (Table 1). The SWCNT-PtNP composite, with an R_ct_ of 88 Ω, combines the properties of both materials. PB lowers the R_ct_ to 42 Ω due to its role as a redox mediator, which enhances electron transfer between the ferri/ferrocyanide redox probe and the working electrode.

For the sensors modified with FL and FL-PB, two distinct semicircles were observed: a high-frequency semicircle (Rf) and a low-frequency semicircle (Rct). This can be explained by the fact that FL forms a thin hydrated film on the carbon working electrode, with internal resistance and capacitance distinct from those of the electrode/electrolyte interface. Similar impedimetric behavior, a high-frequency semicircle corresponding to the internal resistance of the film and a low-frequency semicircle corresponding to Rct at the electrode/electrolyte interface, has been reported for other carbon-based nanomaterials, such as graphene, in electrochemical sensors [42]. For the FL/SPE, Rct decreases only slightly relative to the bare SPE (from 2913 to 2756 Ω), remaining high compared to other sensors due to the film’s lower conductivity, hydrophilic character, and the electron transfer barrier it forms toward the redox probe. For this reason, FL must be combined with other conductive materials, such as the redox mediator PB. FL-PB shows an Rct of 707 Ω, significantly lower than FL alone, but higher than PB alone. The PB precipitated onto the FL enhances the electrocatalytic behavior of the film, although the FL film still exerts an impeding effect.

For the CS/FL-PB/SPE, the Rct is further reduced to 670 Ω compared to FL-PB/SPE. The CS layer facilitates a more uniform deposition of PB on the sensor and ensures ionic conductivity. CS contains protonated amino groups, which can interact electrostatically with the negatively charged ferri/ferrocyanide redox probe, leading to lower resistance. Deposition of the SG film leads to a high Rct of 3867 Ω due to the negative charge of the silanol-based SG matrix, which causes electrostatic repulsion with the ferri/ferrocyanide anions. The SG film may also be thicker and more insulating than the CS-based films, further limiting electronic and ionic transfer at the sensor surface.

The deposition of SG polymeric layers on the SWCNT-PtNP sensors increases the R_ct_ to 174 Ω; however, this value remains substantially lower than that of SG/FL-PB/SPE, indicating that the conductivity of the SWCNT-PtNP composite mitigates the insulating and blocking effects of SG more effectively than FL-PB does. Table 1 summarizes the obtained values of the circuit fitting parameters and goodness-of-fit values for all tested sensors.

The goodness-of-fit values obtained for the circuit-fitted sensors (χ^2^ = 0.022 for SPE and χ^2^ = 0.038 for FL/SPE) confirm acceptable agreement between the experimental data and the selected equivalent circuit models. For the remaining sensors, the three-point graphical method was employed due to the compressed semicircle geometry arising from fast electron transfer kinetics at the conductive nanocomposite surfaces, which limits reliable full circuit fitting.

Overall, the EIS study demonstrates that interfacial charge transfer resistance is governed primarily by the conductive and electrocatalytic properties of the nanomaterials. Among the individual components, PB shows the lowest Rct due to its role as a redox mediator, while the SWCNT-PtNP composite combines high electroactive surface area with catalytic activity, achieving the lowest Rct among the carbon-based nanomaterials used for sensor functionalization. In contrast, FL and SG form resistive films that limit interfacial mass transport, and although PB and CS improve the electron transfer properties of the FL-based layer, their conductivity remains lower than that of SWCNT-PtNP. The variation in Rct across the tested sensors is consistent with the electrocatalytic behavior expected for H_2_O_2_ reduction and with the biosensing performances discussed above, accounting for the higher amperometric sensitivity of SWCNT-PtNP-based sensors. Thus, these EIS results are correlated with the CV and morpho-structural characterization, confirming that SWCNT-PtNP-based (bio)sensors, whether bare or coated with CS or SG as enzyme immobilization matrices, provide the low interfacial resistance and efficient charge transfer required for reliable H_2_O_2_ biosensing applications.

### 3.5. Amperometric Detection of Hydrogen Peroxide and Biomarkers Using (Bio)sensors

The amperometric detection of hydrogen peroxide with SWCNT-PtNP/SPE was optimized with respect to buffer pH and applied potential; the resulting performance parameters are shown in Table 2.

The SWCNT-PtNP-based sensors displayed high sensitivity (224.6 mA M^−1^∙cm^−2^) and a low detection limit of 3.2 μM for H_2_O_2_ at an applied potential of −0.2 V vs. Ag/AgCl, over a wide linear range of up to 28.3 mM at pH 6, while MWCNT-PtNP sensors showed a sensitivity value of 13.23 mA·M^−1^∙cm^−2^ and a low detection limit of 6.8 μM for H_2_O_2_ at an applied potential of −0.2 V.

The high sensitivity of the SWCNT-PtNP sensor is due to the synergistic effect of the two nanomaterials, combining the high surface-to-volume ratio of SWCNTs with the electrocatalytic activity of PtNPs. The SWCNT-PtNP-based sensor shows good sensitivity for H_2_O_2_ detection at pH 6, close to the optimal pH for GOx (5–6) and within the sweat pH range (5–7). In addition, the SWCNT-PtNP sensors can reduce high concentrations of H_2_O_2_ without surface saturation, allowing linear ranges of up to 28 mM.

**Figure 12:**
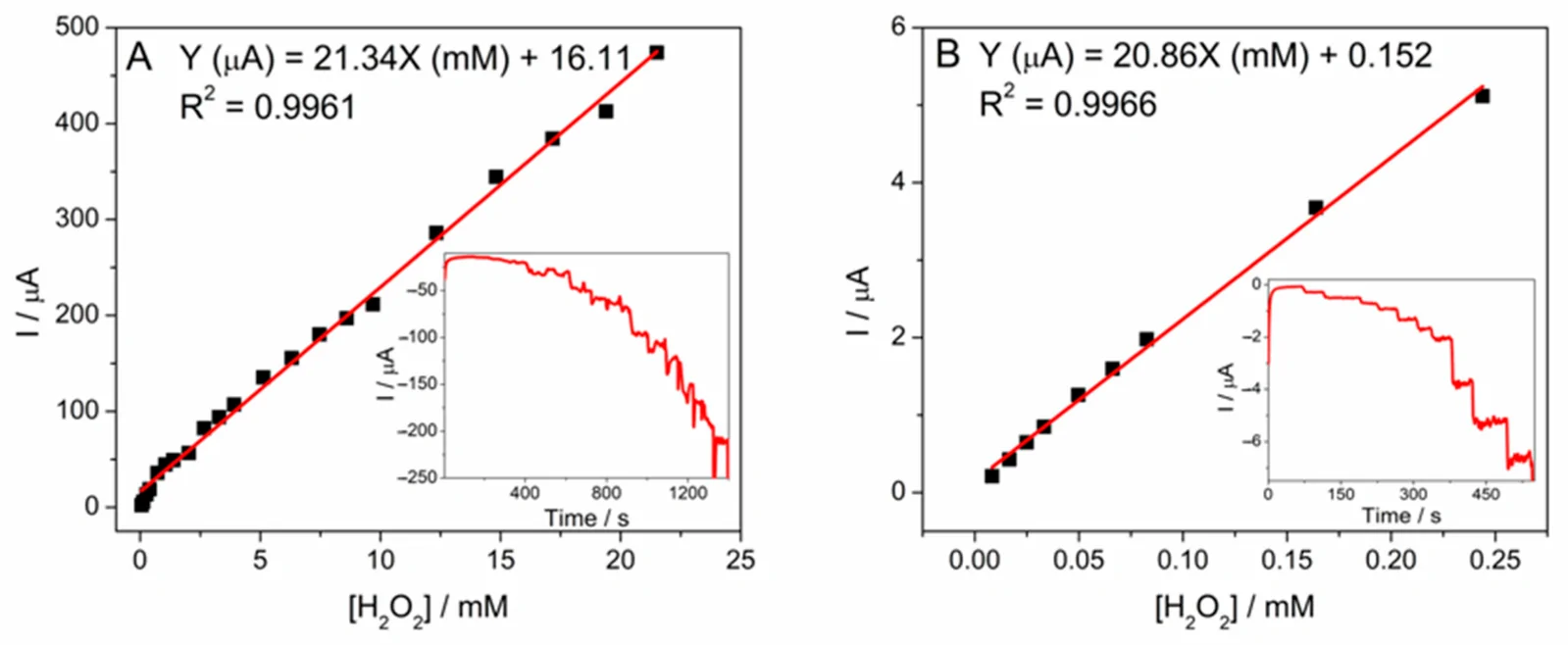
Calibration curves obtained for the amperometric detection of H_2_O_2_ using: (**A**) SWCNT-PtNP/SPE (0.1 M PBS, pH 7, E = −0.2 V vs. Ag/AgCl) and (**B**) FL-PB (0.1 M PBS, pH 8, E = −0.05 V vs. Ag/AgCl).

The calibration curves and the amperometric responses for SWCNT-PtNP sensors at pH 7 and for the FL-PB sensors at pH 8 are presented in Figure 12. SWCNT-PtNP sensors allow detection over a much wider linear range. The FL-PB-modified sensors showed lower sensitivity values and narrower linear ranges, but operated at a lower overpotential. On the other hand, PB, as a redox mediator, reduces the overpotential for H_2_O_2_ reduction to low values (–0.05 V), while FL, which is less conductive than SWCNT and PtNP, provides a hydrophilic surface character and oxygen-containing groups that facilitate PB deposition. FL demonstrates a strong interaction with CS and the GOx, and the composite FL-PB showed a better signal-to-noise ratio compared to SWCNT-PtNP.

Although SWCNT-PtNP presented advantages over FL-PB-modified sensors in terms of higher sensitivity over a wider pH range, FL-PB sensors operate at a significantly lower applied potential (−0.05 V compared to −0.2 V vs. Ag/AgCl), which is an important advantage for biosensor applications, minimizing contributions from electroactive interferents present in biological samples. Thus, both types of sensors were used for biosensor preparation to compare their analytical performance using immobilized enzymes.

**Figure 13:**
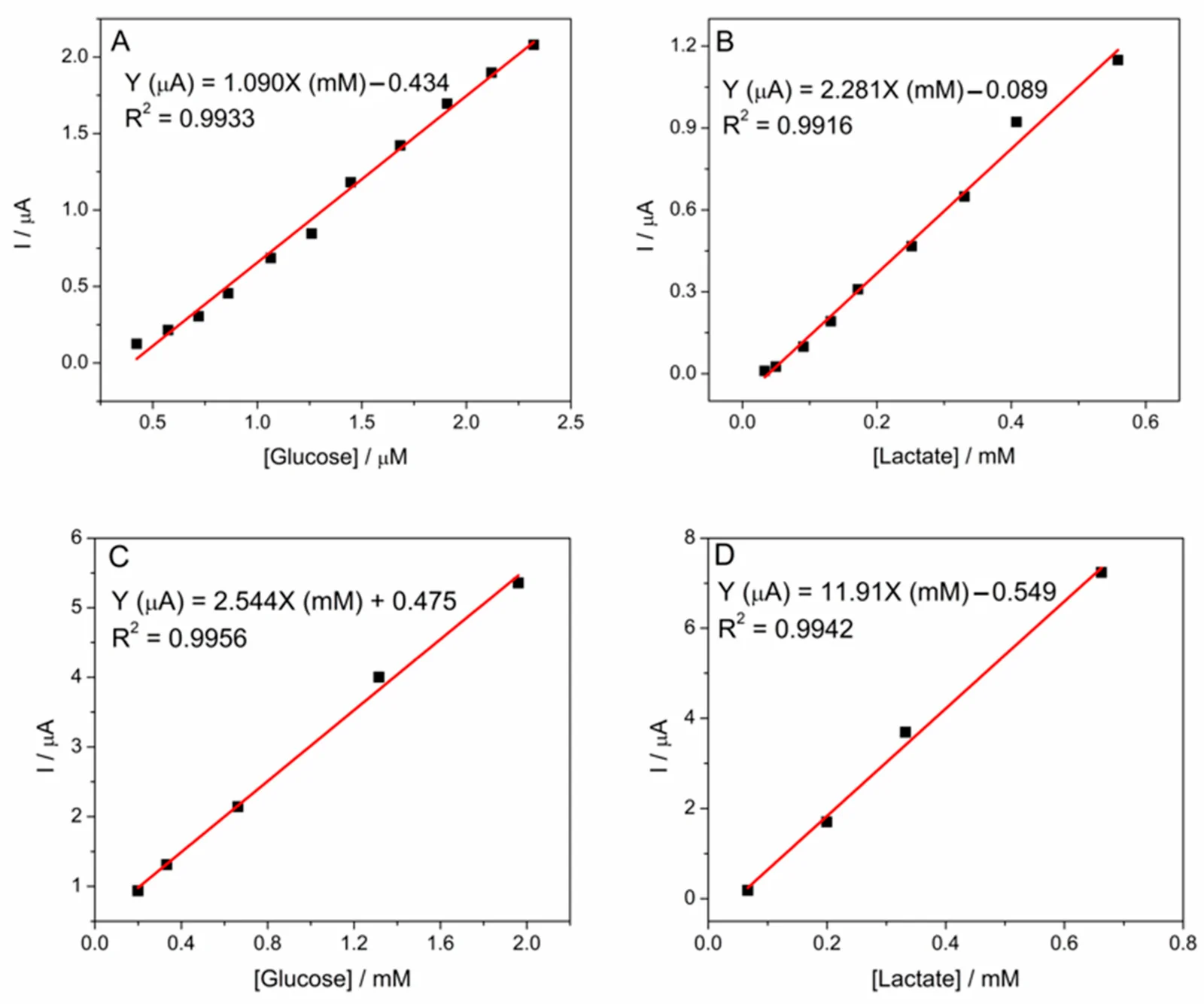
Calibration curves obtained for the amperometric detection of glucose and lactate using: (**A**) GOx-CS/FL-PB/SPE (E = 0 V vs. Ag/AgCl); (**B**) LOx-SG/FL-PB/SPE (E = 0 V vs. Ag/AgCl); (**C**) GOx-CS/SWCNT-PtNP/SPE (E = −0.2 V vs. Ag/AgCl) and (**D**) LOx-SG/SWCNT-PtNP/SPE/SPE (E = −0.05 V vs. Ag/AgCl).

Biosensors based on the oxidase enzymes GOx and LOx were obtained by entrapment in CS and SG matrices on the SWCNT-PtNP/SPEs and FL-PB/SPEs and used for the detection of glucose and L-lactate (Figure 13). The four biosensors, GOx-CS/FL-PB/SPE, LOx-SG/FL-PB/SPE, GOx-CS/SWCNT-PtNP/SPE, and LOx-SG/SWCNT-PtNP/SPE, were characterized by amperometry in 0.1 M PBS pH 7.4 for glucose and L-lactate detection.

For the SWCNT-PtNP biosensors, addition of glucose and L-lactate produced an oxidation current response attributed to enzymatic O_2_ consumption. As the enzymes catalyze substrate oxidation, depletion of dissolved O_2_ reduces the background cathodic current from O_2_ reduction at the PtNPs, resulting in an anodic shift proportional to substrate concentration. For glucose detection, GOx-CS/SWCNT-PtNP/SPE showed the highest specific sensitivity of 20.25 mA∙M^−1^∙cm^−2^ and the lowest detection limit (23.6 µM), with a linear response ranging from 200 to 1960 µM, at an applied potential of −0.2 V. The GOx-CS/FL-PB/SPE biosensor showed a slightly lower specific sensitivity of 8.67 mA∙M^−1^∙cm^−2^ and a detection limit of 55.1 µM glucose, over a wider linear range of 420 and 2300 µM, obtained at an applied potential of 0 V in 0.1 M PBS, pH 7. Amperometric determinations were also performed at pH 5, 6, and 7 for 1 mM glucose. The highest reduction current, 0.778 ± 0.03 µA, was recorded in acetate buffer at pH 5, higher than the 0.145 ± 0.007 µA recorded in PBS at pH 7, indicating higher GOx activity at acidic pH and better biosensor sensitivity.

For LOx-SG/FL-PB/SPE, the highest specific sensitivity value of 18.15 mA∙M^−1^∙cm^−2^ and the lowest detection limit of 26.3 µM for L-lactate were obtained at an applied potential of 0 V in 0.1 M PBS, pH 7. The LOx-SG/SWCNT-PtNP/SPE biosensor detected L-lactate over a linear range of 70–660 μM, with a sensitivity of 94.76 mA∙M−1∙cm−2 and a low detection limit of 5 µM at an applied potential of −0.05 V in 0.1 M PBS, pH 7.

Although SWCNT-PtNP sensors show higher electrocatalytic activity for H_2_O_2_ reduction compared to FL-PB, the amperometric studies showed that biosensor performance is determined mainly by the interactions between the nanomaterial-modified sensor support and the bioreceptor polymeric layer, rather than by the electrocatalytic activity of the underlying sensor alone. Despite its lower sensitivity for H_2_O_2_ reduction, FL-PB-based biosensors showed good enzymatic activity, comparable to that of the SWCNT-PtNP-based biosensors. While the FL-PB composite appears to offer advantages in terms of a better signal-to-noise ratio and operation at lower overpotential (−0.05 V) compared to SWCNT-PtNP (−0.2 V), the combination of significantly higher sensitivity, lower detection limit, and wider dynamic range (up to 28.26 mM) with high sensitivity values of 224.6–198.5 mA·M^−1^·cm^−2^ at sweat-relevant pH values (pH 6–8) justifies the selection of SWCNT-PtNP as the primary platform for biosensor development. Meanwhile, FL-PB shows significantly lower dynamic ranges and lower sensitivity values at acidic pH values. For GOx, the most suitable immobilization matrix was found to be CS. Since GOx has an acidic isoelectric point of 4.2, it carries a net negative charge under neutral or slightly acidic conditions, allowing favorable electrostatic interaction with the positively charged chitosan and enabling stable immobilization. CS also preserves GOx enzymatic activity better than SG and forms a conductive layer together with the nanoparticles and carbon nanotubes, facilitating glucose detection. Table 3 compares the applied potential, sensitivity, and detection limit values of the (bio)sensors developed in this work with those of various amperometric (bio)sensors for glucose and L-lactate detection reported in the literature, with a focus on wearable (bio)sensors used for sweat monitoring.

The response time of the GOx-CS/SWCNT-PtNP/SPE and LOx-SG/SWCNT-PtNP/SPE biosensors, defined as the time required for the amperometric current to reach 90% of its steady-state current value (t90) following substrate addition, was 32 s for glucose and 52 s for lactate, indicating a fast response consistent with the requirements of on-body, near-real-time biomarker monitoring.

Overall, the SWCNT-PtNP and FL-PB-based biosensors allowed the detection of glucose and L-lactate with good sensitivities and relatively low detection limits compared to previously reported biosensors.

### 3.6. Stability, Reproducibility, and Interference Studies for SWCNT-PtNP-Based Biosensors

The long-term stability of the SWCNT-PtNP biosensors was evaluated by carrying out amperometric measurements of 1 mM substrate in 0.1 M PBS, pH 7.4, during a period of 14 days, with the biosensors being kept at 4 °C when not in use. The LOx-SG/SWCNT-PtNP biosensors showed a relatively stable response, having retained 95.4% of the initial amperometric response for 1 mM lactate after 14 days compared to the first day of measurement, while GOx-CS/SWCNT-PtNP/SPE retained 92.6% of the signal intensity for 1 mM glucose.

The operational stability was estimated by measuring the sensor response to 1 mM of substrate in 0.1 M PBS, pH 7.4) over consecutive measurements on the same biosensors. The average current value was 2.49 ± 0.09 μA for 10 successive glucose additions (RSD = 3.4%), recorded with GOx-CS/SWCNT-PtNP/SPE at −0.2 V, while for lactate the average current value was 2.90 ± 0.06 μA (RDS = 2.1%), recorded with the LOx-SG/SWCNT-PtNP biosensor at −0.05 V, with the biosensor retaining over 98% of their initial signal, suggesting good operational stability of the developed biosensors.

**Figure 14:**
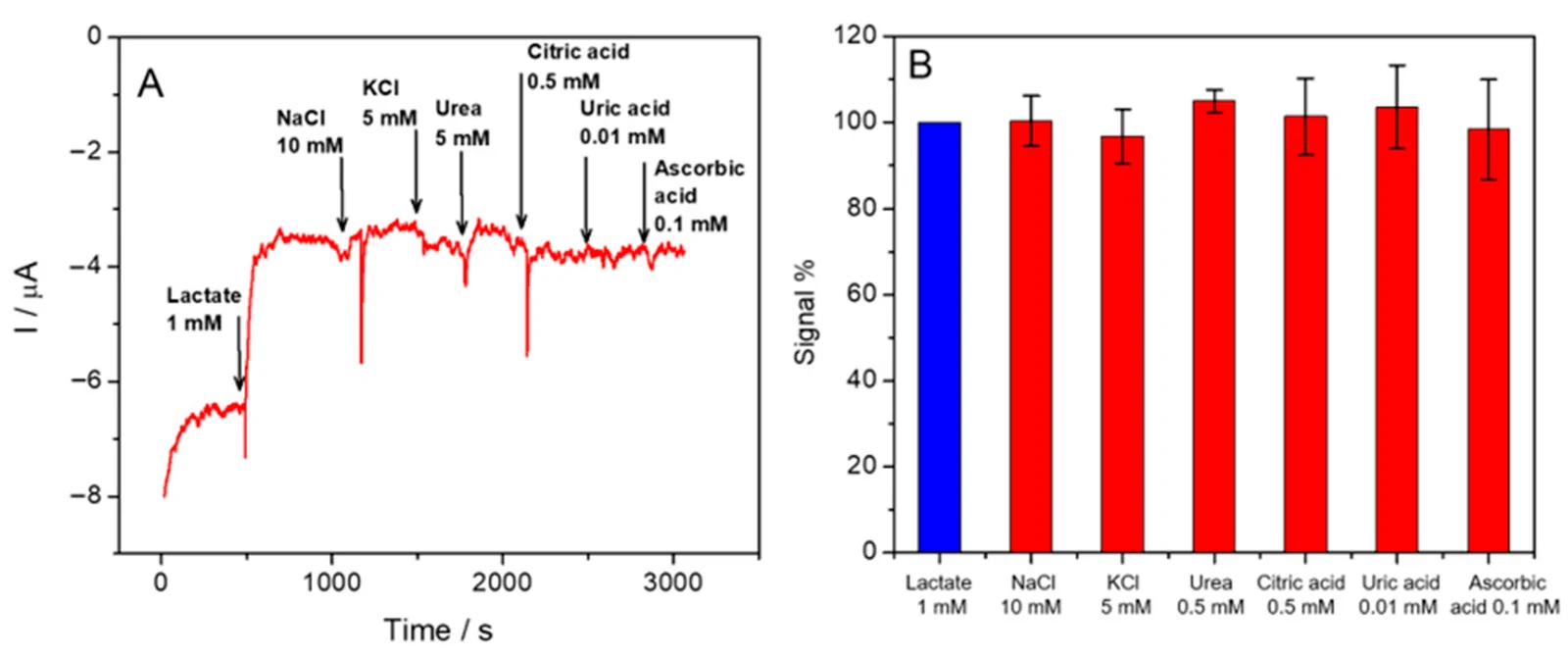
Interference study on the LOx-SG/SWCNT-PtNP/SPE biosensor: (**A**) Amperometric response recorded for sequential additions of L-lactate (1 mM) followed by sweat interferents at physiologically relevant concentrations: NaCl (10 mM), KCl (5 mM), urea (5 mM), citric acid (0.5 mM), uric acid (0.01 mM), and ascorbic acid (0.1 mM). (E = −0.05 V vs. Ag/AgCl, 0.1 M PBS, pH 7.4). (**B**) Relative amperometric response (%) of the biosensor normalized to the lactate signal (100%) (*n* = 3).

Interference studies were carried out using possible interfering compounds found in sweat, such as ascorbic acid, citric acid, uric acid, urea, Na^+^ and K^+^. Figure 14A presents the recorded amperogram and the relative amperometric response (%) of the LOx-SG/SWCNT-PtNP biosensors recorded in 1 mM of substrate, during successive additions of 10 mM NaCl, 5 mM KCl, 5 mM urea, 0.5 mM citric acid, 0.01 mM uric acid, and 0.1 mM ascorbic acid, expressed as the percentage of the initial response for the substrate, in 0.1 M PBS, at −0.05 V.

The LOx-SG/SWCNT-PtNP/SPE biosensor did not show a significant variation in the presence of the interfering species, with the highest variation normalized to the substrate signal being 105.0 ± 2.6% obtained for the addition of 5 mM urea. Thus, the SG matrix provides high selectivity for the detection of lactate with no significant influence from potential interfering compounds in sweat. The selectivity of the GOx-CS/SWCNT-PtNP/SPE biosensor was evaluated under similar conditions. Higher relative responses were observed for several interferents, such as 0.5 mM citric acid (129.9 ± 7%) and 0.1 mM ascorbic acid (138.1 ± 3.8%), attributed to the greater permeability of the CS matrix compared to SG. The need to enhance the selectivity of the GOx-CS configuration will be addressed in future work by further optimization of biosensing layer deposition and incorporation of additional permselective layers.

### 3.7. The Detection of Biomarkers in Real Samples

**Figure 15:**
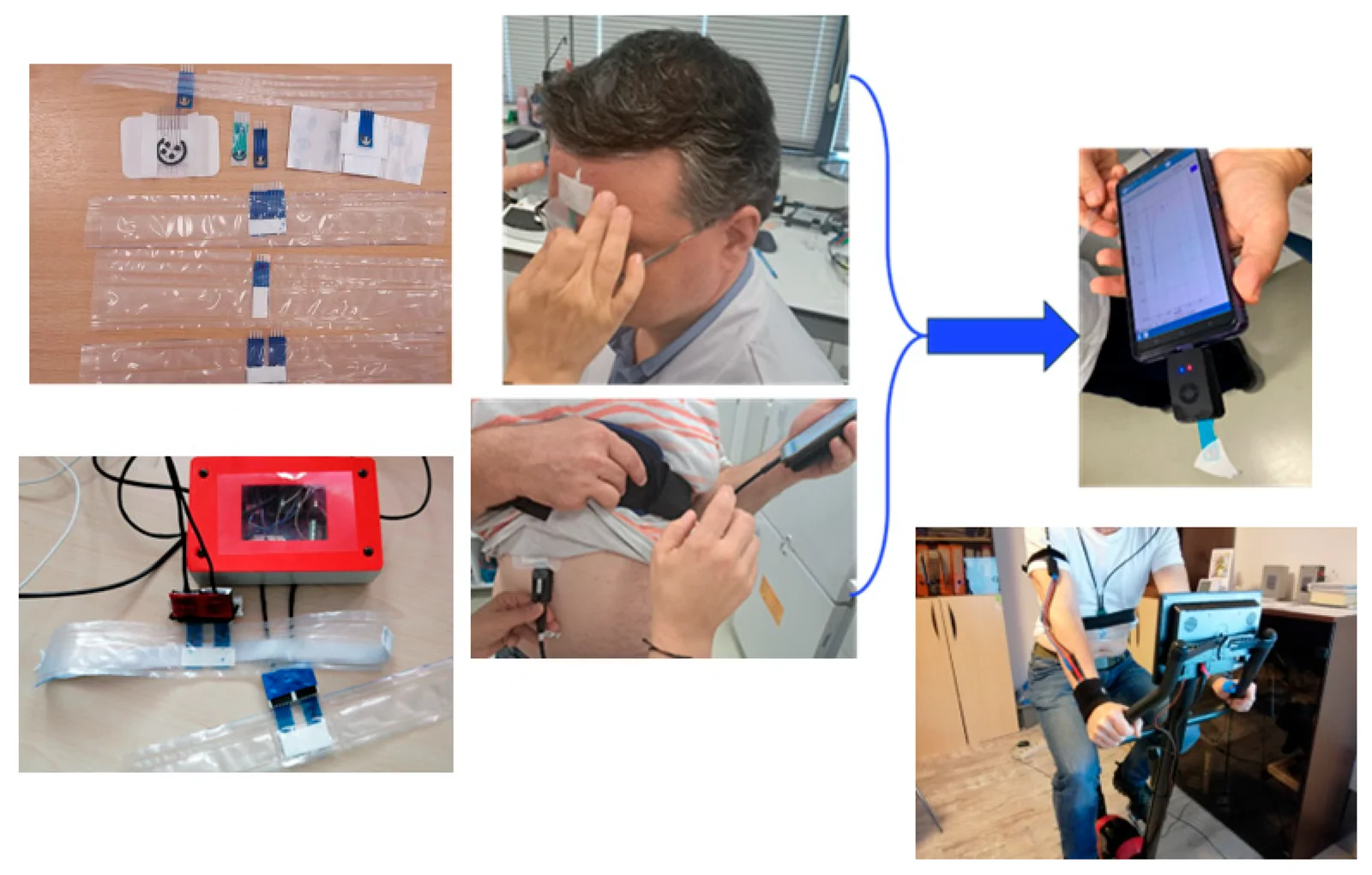
Prototype wearable patches with portable multi-channel electrochemical system for detecting biomarkers from sweat.

The GOx-CS/SWCNT-PtNP/SPE and LOx-SG/SWCNT-PtNP/SPE biosensors were tested for the detection of glucose and lactate in sweat samples using wearable patches, in which the (bio)sensors were integrated onto flexible PVC supports and connected to a portable multi-channel potentiostat (Figure 15). This configuration allowed the biosensors to be fixed onto the skin, and preliminary tests to be carried out to demonstrate device wearability and operation in contact with the skin.

GOx-CS/SWCNT-PtNP/SPE biosensors were used for glucose detection by measuring the amperometric signal first in artificial sweat, and then in artificial sweat spiked with a known amount of glucose. After spiking artificial sweat (3.5 mL) with 250 µL of 1.00 mM glucose (final dilution 1:15), the measured concentration was 0.063 mM, corresponding to a recovery of 94.5%.

Preliminary on-body tests were performed using the wearable patches attached to the abdomen or the forehead, with sweat collected onto a filter paper support in contact with the biosensor surface. However, due to uncontrolled sampling factors, such as surface contamination and non-specific binding, the values obtained were not yet sufficiently reproducible. Standardized sweat collection protocols will be optimized and implemented in future studies.

Several directions for future work have been identified. More replicates using real samples will be carried out, as well as a standardized real-sweat validation study in a larger cohort against a reference method such as HPLC or an enzymatic spectrophotometric assay. A formal immobilization efficiency quantification, by loading optimization across a concentration range, will be carried out for the biosensor fabrication process. A systematic assessment of the mechanical durability of flexible biosensors under repeated bending and stretching will be relevant for long-term use in on-body wearable measurements. Further characterization at different temperatures and ionic strengths will be carried out to ensure functionality over a wide range of conditions. Extended long-term operational and storage stability testing will also be carried out for periods beyond several weeks.

## 4. Conclusions

Polymer-based matrices of CS and SG were used to immobilize enzymatic bioreceptors onto nanomaterial-modified electrochemical (bio)sensors, while SWCNT- and PtNP-based nanomaterials were used to modify the sensor surface, enabling sensitive detection of H_2_O_2_ and sensitive and selective detection of glucose and L-lactate using GOx-CS/SWCNT-PtNP/SPE and LOx-SG/SWCNT-PtNP/SPE biosensors.

Electrochemical studies performed on FL-PB and SWCNT-PtNPs-modified sensors exhibited high electrocatalytic activity toward H_2_O_2_ reduction, facilitating H_2_O_2_ detection at an applied potential of −0.2 V vs. Ag/AgCl, with a specific sensitivity of 224.6 mA·M^−1^·cm^−2^, a linear range up to 28.26 mM, and a low detection limit of 3.2 μM. The GOx-CS/SWCNT-PtNP/SPE and LOx-SG/SWCNT-PtNP/SPE biosensors achieved sensitive detection of glucose and L-lactate at an applied potential of −0.05 V vs. Ag/AgCl, with specific sensitivities of 20.25 and 94.76 mA·M^−1^·cm^−2^, and detection limits of 23.6 and 5.0 μM, respectively.

These biosensors are intended for integration into a multisensing platform for the monitoring of clinical biomarkers, including glucose, lactate, and H_2_O_2_, in sweat samples collected using flexible, wearable patches. The present work serves as an analytical proof of concept for the detection platform and patch integration, with clinical validation being identified as a necessary next step. Future work will also include further optimization of real sweat assays together with validation, studies on the mechanical properties of the flexible sensors, and long-term operational and storage stability testing.

## Figures and Tables

**Table 1:** Values of R_s_, R_ct_, Rf, CPE-n, χ^2^ and OCP for the sensors modified with different nanomaterial layers.

Sensor	Fitting Used	E_OCP_ (V)	Rs (Ω)	Rf (Ω)	Rct (Ω)	CPE-n	χ^2^
SPE	[R(Q[RW])]	0.148	146.18	-	2913	-	0.0220
FL/SPE	[R(RQ)(RQ)]	0.121	152.62	211	2756	-	0.0381
PB/SPE	3 points	0.716	179.23	-	42	0.994	-
FL-PB/SPE	3 points	0.16	201.19	95	707	0.994	-
CS/FL-PB/SPE	3 points	0.16	203.75	54	670	0.997	-
SG/FL-PB/SPE	3 points	0.178	319.1	298	3867	0.997	-
SWCNT/SPE	3 points	0.122	143.0	-	68	0.996	-
PtNP/SPE	3 points	0.116	153.1	-	126	0.998	-
SWCNT-PtNP/SPE	3 points	0.118	166.07	-	88	0.994	-
CS/SWCNT-PtNP/SPE	3 points	0.118	166.85	-	113	0.991	-
SG/SWCNT-PtNP/SPE	3 points	0.136	176.3	-	174	0.991	-

**Table 2:** Electrochemical response characteristics of FL-PB/SPE, SWCNT-PtNP/SPE and MWCNT-PtNP/SPE sensors for hydrogen peroxide detection.

Sensor	Applied Potential (V)	pH	Linear Range (mM)	Sensitivity (mA·M^−1^)	Specific Sensitivity (mA·M^−1^·cm^−2^)	Lod (µM)
FL-PB	−0.05	6	0.008–0.066	4.38	34.82	20.6
		7	0.008–0.066	7.43	59.1	12.1
		8	0.008–0.243	20.86	166.6	4.3
SWCNT-PtNP	−0.2	6	0.07–28.26	28.22	224.6	3.2
		7	0.07–21.51	21.34	169.8	4.2
		8	0.07–12.84	24.94	198.5	3.6
MWCNT-PtNP	−0.2	6	0.07–5.72	13.23	105.3	6.8
		7	0.07–21.92	15.19	120.9	5.9
		8	0.07–12.59	17.27	137.4	5.2

**Table 3:** Comparison of analytical performance parameters for glucose and L-lactate detection of modified biosensors.

Analyte	(Bio)Sensor	E (V)	Linear Range (mM)	Sensitivity (mA·M^−1^)	Specific Sensitivity (mA·M^−1^·cm^−2^)	Lod (µM)	Ref
Glucose	CS + GOx/PtNPs/GCE	0.65	1–13	1.66	23.48	176	[43]
	GR/PANI:rGO/GOx	0.3	0.5–50	2.8	N/A	89	[44]
	GR/PPD/(AuNP)PPCA-GOx	0.2	0.2–150	0.135	N/A	80	[45]
	Nf-GOx/PB/AuNS/GR	−0.05	0.025–1	13.984	N/A	8.8	[46]
	PGOx@MXene/CS/GCE	-	0.03–16.5	3.45	48.98	3.1	[47]
	Wearable (bio)sensors						
	GOx/CBNPs	−0.05	0.005–1.25	-	14.64	4.83	[34]
	AuNFs@CC	-	0.00–8.00	-	63.9	5.18	[48]
	GOx/PEDOT: PSS	0.6	1−10	2.603	-	-	[49]
	MWCNTs-PB	−0.1	0.05−1	-	105.93	4.95	[50]
	GOx-CS/FL-PB/SPE	0	0.42–2.3	1.09	8.67	55.1	This work
	GOx-CS/SWCNT-PtNP/SPE	−0.2	0.2 – 1.96	2.54	20.25	23.6	
Lactate	Au/PB/LOx/CS	−0.1	0–30	N/A	19.13	137	[51]
	LOx/CS/Pt/LSG	0.4	0.2–3	N/A	39.3	110	[52]
	PANI/SPE	−0.15	0.25–10	0.0186	N/A	83	[53]
	SWCNT/NiCo_2_O_4_/HRP/LOx	0.2	0.1–30	0.098	N/A	39.9	[54]
	PU/LOx/PANI/m-PD/SPAuE	0.7	0.20–5.0	1.533	12.17	7.9	[55]
	Wearable (bio)sensors						
	PEDOT/LOx/SPCE	-	0.25–1; 1–40	-	43.42; 0.32	83	[56]
	LOx/PB enzyme–nanozyme	-	0.02–100	-	4.4	20	[57]
	PVC/CHI- LOx/PB/C-MN	−0.1	0.25–35 mM	8.04 × 10^−3^	-	14.8	[58]
	LOx-SG/FL-PB/SPE	0	0.03 – 0.56	2.28	18.15	26.3	This work
	LOx-SG/SWCNT-PtNP/SPE	−0.05	0.070 – 0.66	11.19	94.76	5.0	

Abbreviations: AuNFs@CC—carbon cloth functionalized by Au nanoflowers; CBNPs—carbon black nanoparticles; PANI—polyaniline; PPCA—poly(pyrrole-2-carboxylic acid; MN—microneedle; Nf—Nafion; PB—Prussian Blue; GR—graphite rod; PPD—poly(1,10-phenanthro-line-5,6-dione); AuNS—gold nanostructures, PGOx—GOx polynanogel, PU—polyurethane; m-PD—m-phenylenediamine; SPAuE—screen-printed gold electrode; PMPD—poly-meta-phenylenediamine.

## Data Availability

The original contributions presented in this study are included in the article. Further inquiries can be directed to the corresponding authors.

## Supplementary Materials

The following supporting information can be downloaded at: https://www.mdpi.com/article/10.3390/polym18172150/s1, Figure S1. SEM images of: (A) SG/FL-PB/SPE, (B) SG/SWCNT-PtNP/SPE, (C) CS-GOx/SWCNT-PtNP/SPE, and (D) SG-LOx/SWCNT-PtNP/SPE; Figure S2. Full-range FTIR spectra (4000-500 cm-1) for: (A) FL (a), PB (b), and FL-PB (c); (B) SWCNT (d), PtNP (e), SWCNT-PtNP (f); (C) CS powder (g), CS in 2% acetic acid solution (h), GOx (i), GOx-CS (j); and (D) SG (k), LOx (l), and LOx-SG (m); Figure S3. Schematic representation of the equivalent circuits used for EIS fitting: (A) [Rs(Qdl[RctW])], and (B) [Rs(RfQf)(RctQdl)].
