# Supplementary material for: A Flexible Wearable Multisensing Patch Integrating SWCNT-PtNPs Nanocomposites for Non-Invasive Clinical Biomarkers Monitoring in Sweat
Source: Polymers (Basel). 2026 Sep 2;18(17):2150. doi: 10.3390/polym18172150 (PMC13568237; doi:10.3390/polym18172150)
Supplement: Supplementary file 1 [file polymers-18-02150-s001.zip › polymers-4515006-supplementary.pdf]

Supplementary Materials

A flexible wearable multisensing patch integrating SWCNT-PtNPs nanocomposites for non-invasive clinical biomarkers monitoring in sweat

Lucian-Gabriel Zamfir [1,*], Petru Epure[2], Cătălina Gîfu [1], Iuliana Răut [1], Mariana Constantin [1], Cristina Firincă [1], Nicoleta-Olguța Corneli [4], Mihaela Doni [1] and Ana-Maria Gurban [1,*]

1 National Institute for Research and Development in Chemistry and Petrochemistry-ICECHIM, Department of Biotechnology, 202 Spl. Independentei, 060021, Bucharest, Romania;

2 EPI-SISTEM SRL, Livezii 17A, Sacele, Brasov, Romania

3 National Institute for Medical-Military Research and Development Cantacuzino, 103 Spl. Independentei, 050096, Bucharest, Romania;

* Correspondence: lucian.zamfir@icechim.ro; ana-maria.gurban@icechim.ro;

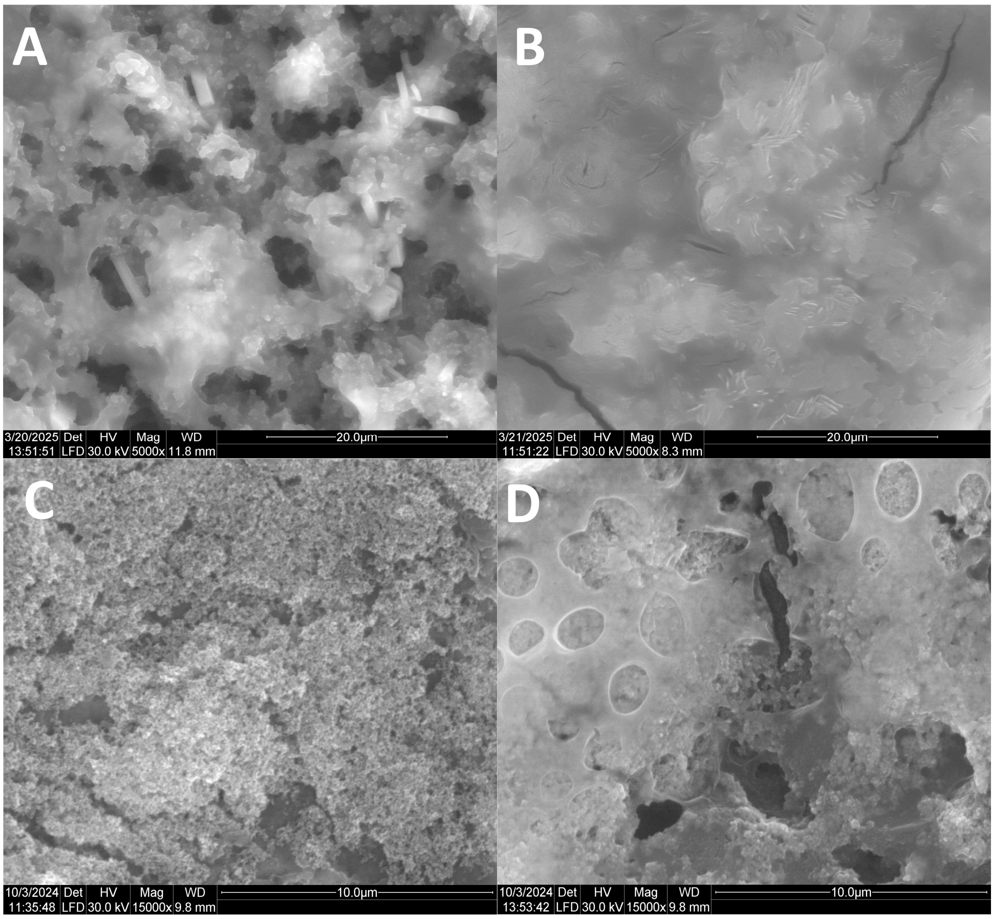

Figure S1. SEM images of: (A) SG/FL-PB/SPE, (B) SG/SWCNT-PtNP/SPE, (C) CS-GOx/SWCNT-PtNP/SPE, and (D) SG-LOx/SWCNT-PtNP/SPE.

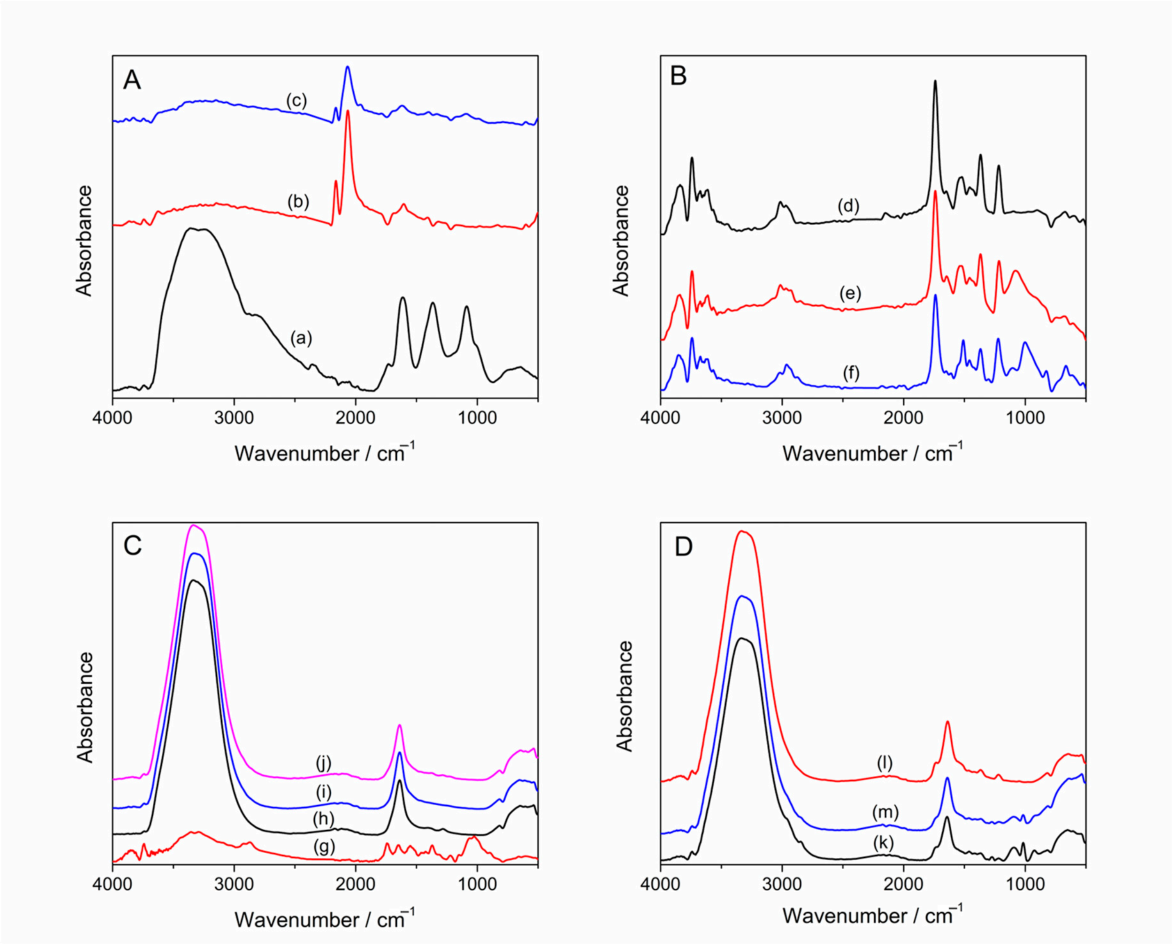

Figure S2. Full-range FTIR spectra (4000-500 cm^{-1}) for : **(A)** FL (a), PB (b), and FL-PB (c); **(B)** SWCNT (d), PtNP (e), SWCNT-PtNP (f); **(C)** CS powder (g), CS in 2% acetic acid solution (h), GOx (i), GOx-CS (j); and **(D)** SG (k), LOx (l), and LOx-SG (m).

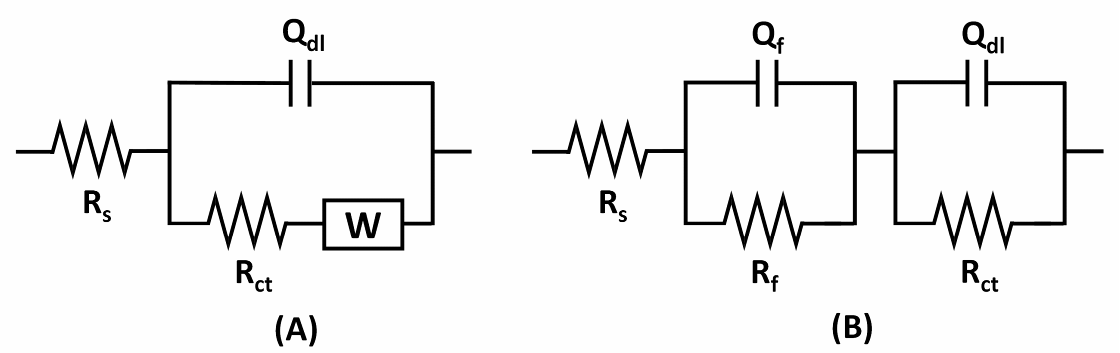

Figure S3. Schematic representation of the equivalent circuits used for EIS fitting: (A) [Rs(Qdl[RctW])], and (B) [Rs(RfQf)(RctQdl)].
